# Secondary Immunodeficiency in Hematological Malignancies: Focus on Multiple Myeloma and Chronic Lymphocytic Leukemia

**DOI:** 10.3389/fimmu.2021.738915

**Published:** 2021-10-25

**Authors:** Alessandro Allegra, Alessandro Tonacci, Caterina Musolino, Giovanni Pioggia, Sebastiano Gangemi

**Affiliations:** ^1^ Division of Hematology, Department of Human Pathology in Adulthood and Childhood “Gaetano Barresi”, University of Messina, Messina, Italy; ^2^ Clinical Physiology Institute, National Research Council of Italy (IFC-CNR), Pisa, Italy; ^3^ Institute for Biomedical Research and Innovation (IRIB), National Research Council of Italy (CNR), Messina, Italy; ^4^ School of Allergy and Clinical Immunology, Department of Clinical and Experimental Medicine, University of Messina, Messina, Italy

**Keywords:** immunodeficiency, infection, multiple myeloma, chronic lymphocytic leukemia, vaccination

## Abstract

Secondary immunodeficiency is reported in most patients with hematological malignancies such as chronic lymphocytic leukemia and multiple myeloma. The aim of our review was to evaluate the existing literature data on patients with hematological malignancies, with regard to the effect of immunodeficiency on the outcome, the clinical and therapeutic approach, and on the onset of noninfectious complications, including thrombosis, pleural effusion, and orofacial complications. Immunodeficiency in these patients has an intense impact on their risk of infection, in turn increasing morbidity and mortality even years after treatment completion. However, these patients with increased risk of severe infectious diseases could be treated with adequate vaccination coverage, but the vaccines’ administration can be associated with a decreased immune response and an augmented risk of adverse reactions. Probably, immunogenicity of the inactivated is analogous to that of healthy subjects at the moment of vaccination, but it undertakes a gradual weakening over time. However, the dispensation of live attenuated viral vaccines is controversial because of the risk of the activation of vaccine viruses. A particular immunization schedule should be employed according to the clinical and immunological condition of each of these patients to guarantee a constant immune response without any risks to the patients’ health.

## Introduction

### General Considerations on Secondary Immunodeficiency: Immunity and Infections

It is possible to classify immunodeficiency syndromes in different manners. A possibility is to separate primary and secondary immunodeficiency. Primary conditions stem from a hereditary alteration of the immune system; this type of disease being usually divided into alterations involving the T-cell system, the B-cells or both B- and T-cells. Generally, they are evident soon in life (1). Secondary immunodeficiencies (SID) happen more often than the previous ones and generally appears in elder patients as an effect of an external factor, such as human immunodeficiency virus (HIV) infection, malaria, severe liver disease, uremia, malnourishment, splenectomy, diabetes mellitus, cancer and cancer treatment (2). A different cause of SID might be nephrotic syndrome or a protein losing enteropathy with an extreme loss of immunoglobulin. Finally, severe burns may also decrease the immune response.

However, SID generally have a composite pathogenesis correlated to both the principal disease and the effects of its pharmacological treatment (2). Disease‐connected SID include solid tumors, chronic and acute lymphoproliferative and myeloproliferative disorders (1).

The clinical effect of SID may extend from a moderately relevant infection vulnerability to a more serious condition distinguished by repeated pulmonary infections, viral or fungal opportunistic infections (1, 3). Moreover, in this type of patient, the diffusion of multidrug-resistant organisms (MDRO), specifically multidrug-resistant gram-negative bacteria, vancomycin-resistant enterococcus and methicillin-resistant staphylococcus aureus augment the incidence of severe infections and mortality. Subjects with disease- or treatment-correlated SID are particularly at risk for lethal infections provoked by MDRO (1). Finally, a frequent reason of impaired immune response is the same infectious condition, which alters the activity of lymphocytes. Finally, the extensive use of cytotoxic treatments and immunosuppressive drugs in tumor subjects can cause a condition of grave SID (4). Therapy-related SID can also happen due to the use of anti‐inflammatory and biological medications, especially in transplanted patients (5).

The aim of this review is to evaluate the onset of secondary immunodeficiency in patients with hematological malignancies with a particular focus on multiple myeloma (MM) and chronic lymphatic leukemia (CLL). We will try to evaluate the mechanisms of onset of immunodeficiency, the effectors involved, the effects on survival, the occurrence of complications and possible therapeutic interventions.

## Characteristics of Secondary Immunodeficiency in Patients With Multiple Myeloma

### Epidemiology and Incidence

Multiple myeloma (MM) is a neoplasm of bone marrow plasma cells that provoke intense immunodeficiency (6). Significant progresses in anti-myeloma treatment have enhanced survival (7–11); however, infections cause a fatal outcome in one out of five subjects with MM (12). The risk of getting an infection is highest in the first 90 days after diagnosis, with a third of subjects experiencing severe bacterial diseases that are the cause of about half of early mortality (13, 14).

In MM subjects, the hazard ratios of getting diseases such as septicemia, meningitis, or pneumonia have been shown to be 7.7-, 15.6-, 16.6- and 7.7-fold, respectively, with respect to controls (15). In a study, of the 412 MM subjects studied, 37.4% were reported to develop at least one infectious event, and an incidence of 244 infectious events were recorded. The more frequent sites of infection were the lung and the genitourinary apparatus, while the more frequent infections were bacterial, followed by viral, and *Escherichia coli* resulted in being the most common microorganism. However, in 65.5% of cases, the organism was not identified. Infection was the principal reason of death in 6.3% out of all subjects (16). In MM subjects, risk elements of infection were Durie-Salmon stage IIIB, neutropenia, sex (female), augmented serum creatinine, bad performance status and the presence of a catheter indwelling (17).

Particularly interesting are the data relating to the relationship between MM and hepatitis viruses.

Recently, a study demonstrated an augmented risk of reactivation of HBV after daratumumab administration (18). However, other studies appear to demonstrate a two-way relationship between hematological disease and viral infections. Patients with hepatitis virus infection were reported to have a greater risk of MM, perhaps *via* interference with immunosurveillance (19). In several reports, the frequency of HCV infection in MM has been demonstrated to be greater than in a control population (20), and in a study performed by Duberg and colleagues, there was a relevant argument in the risk of MM onset in subjects with HCV infection for more than 15 years (21). However, a similar correlation was not reported in other studies (22, 23).

A population particularly at risk of infection is that of the MM subjects undergoing transplantation. The degree of hazard of infection in patients undergoing autologous HCT may be distinguished into two phases: pre-engraftment, which is characterized by the occurrence of neutropenia and mucositis, and post-engraftment, which is correlated to a gradual improvement of cell-mediated immunity (24). Before the engraftment, infections essentially involved cellulitis, bacteremia, and gastrointestinal infection (*Clostridium* difficile) (24). The factors favoring the onset of infections prior to starting conditioning treatment comprise kidney insufficiency, smoking, and iron overload. However, the length of neutropenia is the principal risk element after the conditioning regimen is administered (25, 26). The relationship between the iron burden and a greater risk of infectious diseases has been repeatedly described, especially after autologous or allogenic bone marrow transplantation (27, 28).

T-cell normalization happens gradually and is driven by the MM remission condition, the conditioning treatment, the use of radiation treatment, and the type of anti-myeloma treatment (24). Fatal delayed infection after bone marrow transplantation can be provoked by varicella-zoster virus, cytomegalovirus, *C. difficile*, and *Pneumocystis jirovecii*, which provokes pneumonia (29).

### Causative Mechanisms of Secondary Immunodeficiency in Patients With Multiple Myeloma

**Figure 1:**
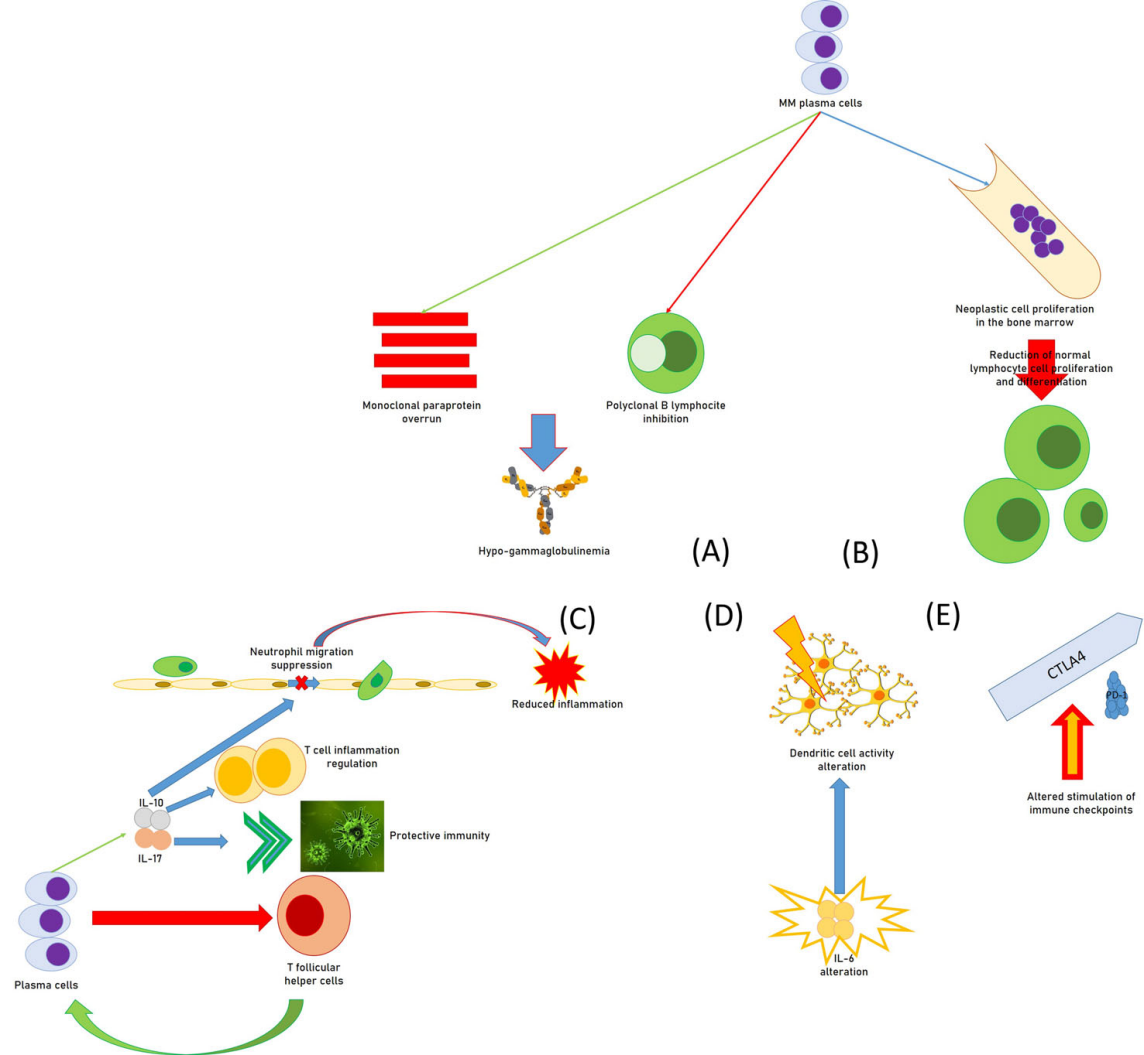
Causal moments of immunodeficiency in patients with multiple myeloma: **(A)** functional hypogammaglobulinemia; **(B)** deficit of the proliferative attitude of lymphocytes; **(C)** regulation of immune functions by plasma cells, with the reduction of T follicular helper cells, the promotion of IL-17, increasing protective immunity against some infections, the source for IL-10, in turn regulating T-cell-related inflammation and suppressing neutrophil-related inflammation; **(D)** alteration of the activity of the dendritic cells by huge immune alterations, including IL-6; **(E)** alteration of the immune checkpoints (altered stimulation of CTLA-4 and PD-1) provoking immunodeficiency.

Regarding the mechanisms by which MM patients develop secondary immunodeficiency, all of the effectors of the immune system can be involved in the genesis of SID (**Figure 1**). For instance, an overrun of monoclonal, non-efficient paraprotein and inhibition of polyclonal B lymphocytes by MM plasma cells cause hypogammaglobulinemia, while the proliferation of neoplastic cells in the marrow prevents normal hematopoiesis and determines a reduction in the production of functionally active lymphocyte cells. In fact, in addition to their action in the production of antibodies, plasma cells exert an important role in the regulation of immune functions. For instance, they operate reducing T-follicular helper cells that are essential for the same production of plasma cells in immune reactions that are T-dependent (30). Moreover, CD138hi plasma cells/plasmablasts generate IL-17, a cytokine able to induce protective immunity against *Trypanosoma cruzi* infection (31).

Furthermore, a study suggested that CD138hi cells can be a relevant source of IL-10 which can regulate T-cell–related inflammation (32), while MM cell lines seem able to produce IL-10 (33).

In a report, IL-10 amounts increased with MM progression in patients, and the cytokine quickly suppressed neutrophil migration and reduced neutrophil-related inflammation in an animal experimental model of autoimmune disease (34). It is also important to point out that neutrophils are essential to produce a well-organized immune response against gram-positive bacteria, which are the most common reason for grave infections in subjects with MM and lupus (35).

Moreover, MM patients with a poor long-term survival prognosis had less-replicated cytotoxic T-cell clones, a minor amount of Th17 cells, and a greater number of T-regulatory cells (Tregs) (36). In addition, DC activity might be modified by intense immune alterations of diverse immune components including IL-6, Transforming growth factor-β, and PGE2 within MM bone marrow milieu (37, 38). Altered stimulation of cytotoxic T-lymphocyte associated protein 4 (CTLA-4) and programmed cell death protein-1 (PD-1) immune checkpoints also provoke unwanted immunodeficiency in MM patients (39, 40). B7-H1 is an immunoglobulin-like immunosuppressive molecule largely present in human tumors. It sends an inhibitory signal to its counter-receptor, PD-1, on T cells, thus provoking T-cell inhibition, MM progress, and chemoresistance (41, 42). In MM patients, the existence of a milieu of immunosuppressive activity has a fundamental role in T-cell immunodeficiency. Several immunosuppression elements are produced by the bone marrow cells, which alter innate and adaptive immune responses (43).

Furthermore, the phenomenon of T-cell exhaustion was reported in many solid cancers and hematological malignancies. This condition is due to a deficiency of proliferative aptitude, reduction of cytotoxicity, an altered delivery of different effector cytokines, such as interleukin 2 and interferon-gamma, and the augmented apoptosis of exhausted T cells (44). An increased presence of T-cell inhibitory receptors is another feature of exhausted T cells (45). For instance, T-cell immunoglobulin and mucin-domain-containing-3 (Tim-3) with its ligand galectin-9 cause CD8+ T-cell alteration and exhaustion in several hematological malignancies (46–48). In recent times, increased inhibitory receptors such as lymphocyte activation gene-3 (LAG-3) on CD4+ and CD8+T cells have been described in peripheral blood from MM subjects before and after autologous transplantation (49).

These aspects have been clarified in a recent study (50), where a relevant increase in both PD-1+CD57+ and Tim-3+CD57+CD3+ T cells and PD- 1+Tim-3+CD3+ T cells was described in blood from MM subjects with respect to controls, and the expression was essentially in the CD8+ T-cell compartment. Moreover, a substantially greater fraction of PD-1+CD3+ T cells was discovered in bone marrow with respect to peripheral blood in MM subjects.

Finally, albeit found in a small number of patients, the finding that after a complete remission, patients exhibited a reduction of the numbers of either PD-1+ or PD-1+Tim-3+ T cells in diverse T-cell compartments in both bone marrow and peripheral blood is extremely interesting (50).

### Influence of the Therapeutic Treatment of Multiple Myeloma on the Onset of Secondary Immunodeficiency

Recent reports have proposed that new MM drugs and elevated doses of steroids can determine a permanent risk of infection, even in subjects whose myelomatous disease is well controlled. This infection risk seems to persist high throughout the first year after diagnosis (50). With the use of immunotherapy, therapeutic protocols have an even more pronounced immunosuppressive effect. Moreover, these treatments are maintained for long time, increasing the risk of infection-correlated death.

With regards to SID treatment-related, alterations of innate immunity are crucial (51). Innate immune cells include granulocytes, macrophages, monocytes, and dendritic cells. Numerous groups of pathogen recognition receptors (PRRs), including NOD‐like receptors, toll‐like receptors, RIG‐I‐like receptors, and cytosolic sensors for DNA, are recognized to have a relevant effect in host defense (52, 53). Signals from various PRRs determine the passage of NF‐κB into the nucleus, leading to a cytokine inflammatory response (54), but MM patients take drugs which block nuclear passage of NF‐κB.

When dexamethasone is used, infection provoked by a decline of cell-mediated immunity becomes more probable, which may be realized in the form of mucosal candidiasis or viral or bacterial infections (55, 56). Nevertheless, a 2005 study demonstrated that the overall infection frequency after dexamethasone was comparable to that observed after melphalan plus prednisone (55), even though minor cumulative dosages of dexamethasone considerably reduce the occurrence of grade 3–4 infection, with respect to the usual high dose (57) (**Table 1**).

**Table 1:** Influence of the therapeutic treatment of multiple myeloma on the onset of infections.

DRUG	Effect on the risk of infection	Mechanisms	Ref.
Dexamethasone	Increased	Decline of cell-mediated immunity	(55, 56)
Thalidomide	None		(60, 61)
Lenalidomide	None	Neutropenia	(63, 64)
Bortezomib	Increased	Neutropenia Reduction on T cell growth Decrease of NK cells Depression of dendritic cells Change of cytokine production	(25, 58, 59, 65, 66, 68, 69, 71)
Bisphosphonates	Increased	Impairment of neutrophils function	(73)

Thalidomide, a drug with immunomodulatory activity, is not appreciably myelotoxic (28) and exerts both immunomodulatory and immunosuppressive actions on T cells (58, 59). In any case, thalidomide does not seem to augment the risk of infection in MM subjects, as reported in a controlled study of dexamethasone *versus* dexamethasone plus thalidomide (60). This was also reported in a second study that compared melphalan plus prednisone *versus* melphalan plus prednisone with thalidomide (61).

Lenalidomide, a derivative of thalidomide, has more powerful stimulatory actions on CD4+ and CD8+ T cells than thalidomide (62). In a study performed on newly diagnosed MM subjects treated with lenalidomide plus dexamethasone, severe neutropenia (grade III-IV) was infrequent (12%), and only 1 subject presented severe infection (63). In a different report, 68% of subjects on melphalan plus prednisone and lenalidomide presented neutropenia, but only 9% displayed severe infection, a percentage similar to that reported in subjects treated with melphalan plus prednisone (64).

Bortezomib, a proteasome inhibitor, is capable to provoke neutropenia (65); reduction in T cell growth (58, 66); decrease of NK and CD8+ T cells (59); alteration of NK and CD8+T cells function (59); suppression of dendritic cells (67, 68) and changes in cytokine production (69).

Particularly, Bortezomib exerts powerful immunosuppressive consequences on T cells (70, 71). Nevertheless, a controlled clinical study did not report an augmented risk of infection, and the percentages of varicella zoster virus infection were analogous to those of the controls after the introduction of varicella zoster virus prophylaxis (71).

Finally, Bisphosphonates can determine local decrease of immune response with an unidentified system (72), although an impaired neutrophil function may partially contribute to an increased susceptibility to infections (73). Osteomyelitis of the jaw progresses into osteonecrosis (74), *via* diverse mechanisms of both infectious and antiangiogenic types (75, 76). A state of immunodeficiency can favor the onset of septic states capable of inducing osteonecrosis.

The assessment of susceptibility to infections and the risk of infectious complications should be evaluated in each patient before the choice of the therapeutic protocol. This could prevent or limit the onset of an immunodeficiency condition secondary to therapy which could lead to a bad outcome.

### Prophylaxis and Therapy of Secondary Immunodeficiency in Patients With Multiple Myeloma

MM patients should be submitted to a risk evaluation preceding the beginning of anti-myeloma therapy on the base of disease- and patient-correlated elements. Multiple myeloma-related aspects able to modify therapy choices include cytogenetic alterations, lactic dehydrogenase concentrations, stage disease, augmented serum Beta2-microglobulin amounts, and others (77). Assessment of host-correlated elements comprises a meticulous anamnesis with specific emphasis on previous infections, a physical evaluation, and a complete evaluation of liver, kidney, pulmonary, and metabolic functions (78).

As for an adequate prophylaxis, the administration of intravenous immunoglobulins (IVIg) for 6–12 months decreased the possibility of severe infections (grade A recommendation, level 1b evidence) (79).

In a recent study, 45 of 295 MM subjects with IgG <5 g/L were defined as SID patients. These patients mostly had repeated infections, particularly pulmonary bacterial infections. The median survival time was considerably shorter in MM subjects with SID (24 *vs* 66 months). More importantly, the multivariate and univariate analysis revealed that IgG <5 g/L was an independent prognostic factor for MM patients (80).

Reduced amounts of specific antibodies against diverse, bacterial, viral, and fungal agents have been reported in patients (81). Nevertheless, not all MM subjects with reduced immunoglobulin levels have infectious problems; moreover, severe infections can occur in the lack of hypogammaglobulinemia. These events could be due to neutropenia or T cell inhibition. B-cell dysfunction augments gradually from monoclonal gammopathy of undetermined significance (MGUS) to Waldenstrom macroglobulinemia to MM (79, 81).

Although the outline of bacterial complications is analogous to that reported in other conditions characterized by neutropenia, most infections in MM subjects are not correlated to reduced neutrophil counts but result from diverse conditions, such as reduced defense capacity of the biological barrier and decreased immune response. In any case, there are only few studies on antimicrobial prophylaxis in MM patients, and for this reason there is some uncertainty for the use of antimicrobial prophylaxis in MM subjects (82).

However, in this setting, preventive treatment with antiviral or antibiotic drugs has been broadly employed (83). Continuous dispensation of antiviral drugs such as acyclovir, famciclovir, or valacyclovir confirmed to be efficient in avoiding herpes zoster virus infections in subjects treated with bortezomib (84). For patients undergoing to autologous transplantation, prophylactic administration of levofloxacin provoked a 27% reduction in infections as well as a 31% reduction in neutropenia or fever (85). Nevertheless, the efficacy of prophylactic antibiotic was reduced by the augmented presence of resistant strains and drug toxicity. Moreover, the administration of antibiotics might provoke unwanted drug interactions and antagonistic effects. In fact, the drugs used in patients with MM, such as bortezomib, corticosteroids, and thalidomide, may be P450 competitors or inducers (86, 87). Therefore, it is important to pay close attention when employing P- 450–related antimicrobial drugs (88).

For these reasons, antibiotics should only be administered for high infection-rate conditions, such as induction treatment, progression, or refractoriness (89). In fact, adding of prophylactic levofloxacin to induction therapy during the first 90 days of treatment drastically decreased febrile occurrences and fatal outcomes with respect to placebo (88).

Finally, there may be supplementary factors motivating the advantages of levofloxacin prophylaxis other than a decrease in infection, and a modification in the microbiome might participate to a decrease in inflammation and an increase in the well-being of these subjects (7).

## Characteristics of Secondary Immunodeficiency in Patients With Chronic Lymphocytic Leukemia

Chronic lymphocytic leukemia (CLL) is a hematological disease with an unpredictable prognosis. This was partially due to the augmented occurrence of infections, and these complications are the main cause of morbidity and death in CLL subjects (90). Generally, infections are of bacterial nature and tended to occur in the lung; however, they can also be located in the gastrointestinal tract, or the skin (91).

Numerous effectors of the immune response are altered in CLL patients, comprising both the alterations of the immune response correlated to the disease itself and the effects of immunosuppression deriving from chemoimmunotherapy of CLL (92, 93).

CLL subjects were described as exhibiting antibody generation reduction (94, 95), and Bussel and Cunningham-Rundles (96) suggested that they might profit from antibody treatment (96).

**Figure 2:**
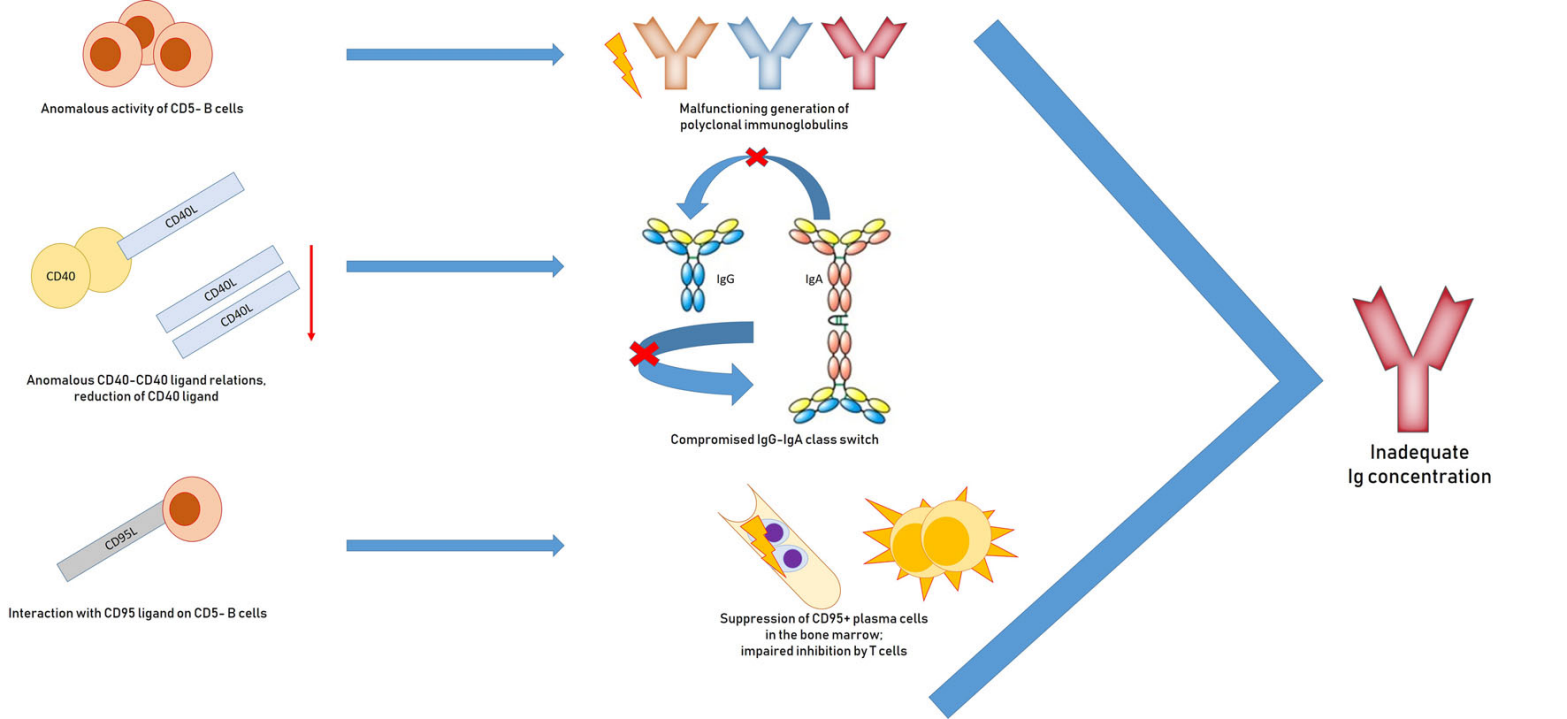
Pathogenesis of hypogammaglobulinemia in patients with chronic lymphocytic leukemia: impaired generation of polyclonal immunoglobulins due to inadequate activation of CD40, suppression of plasma CD95 + cells, impaired inhibition by T cells.

Reasons for inadequate immunoglobulin concentrations include malfunctioning generation of polyclonal immunoglobulins and anomalous activity of non-neoplastic CD5− B cells; a IgG and IgA class-switch *via* anomalous CD40–CD40 ligand relations and reduction of CD40 ligand; suppression of CD95+ plasma cells in the bone marrow through interaction with CD95 ligand on CD5-B cells; disproportionate inhibition by T-cells; and iatrogenic myelosuppressive treatment (97, 98) (**Figure 2**).

The occurrence of reduced gammaglobulinemia in CLL patients is more marked with advanced-stage disease and linked with patient age and associated pathologies (91). This occurs in CLL subjects with mutated and unmutated immunoglobulin heavy chain genes (99–101).

A 2006 study reported the existence of considerably lower functional antibody amounts against 16 of the 19 serotypes evaluated in old CLL subjects with respect to a group of unvaccinated control subjects (101). As for pneumococcal serotypes, protecting concentrations of antibodies were reported in only 3 of 12 serotypes, in contrast to 9 out of 12 within the control group. This finding suggests that the specific antibody reduction described in CLL patients is correlated to the disease itself and not just a consequence of immune-senescence secondary to aging.

Furthermore, 79% of CLL subjects with a normal IgG amount still had reduced specific antibody reactions to pneumococcus, revealing that IgG evaluation alone is not adequate to recognize CLL subjects who are at increased risk of infection (102). Then, even though British Committee for Standards in Hematology guidelines for the treatment of CLL suggests assessment for total immunoglobulin concentrations as a method of recognizing subjects at risk of infection (103), this approach might be unsuccessful for detecting those subjects with a regular IgG level that have inadequate functional antibody amounts.

Evaluation of IgG antibodies to distinctive antigens results from verified contacts or immunization with specific proteins such as tetanus, diphtheria, or hepatitis B, or polysaccharides such as Salmonella typhi Vi. Specific tests are also available for specific antibodies to herpes simplex virus, Epstein‐Barr virus, varicella‐zoster virus, and pneumococci. However, in some cases, such as for pneumococcal antibodies, analyses are remarkably inconstant, and evaluation can be troublesome (104).

Complement amounts are also reduced in CLL subjects, especially the C3b fraction, which augments the occurrence of repeated bacterial infections. However, there are also alterations in the stimulation, connection and presence of the complement receptor 1 (CR1) and CR2. Fust et al. reported alterations in the classical complement system in CLL subjects, with a decrease in C1 and C4 amounts in more than 50% of subjects examined (105).

CLL subjects also experience abnormal cellular immunity. Generally, the amount of T helper cells is reduced while the number of T suppressor cells is augmented. Furthermore, CD3+CD8+ T cells generate a decreased quantity of IL-2 and an augmented quantity of interferon and tumor necrosis factor (106–108).

Alterations in phagocyte activity have also been described. Defects in the production of digestive enzymes can involve several types of cells such as neutrophils, monocytes, and lymphocytes. These deficiencies comprise lysozyme, b-glucuronidase, and myeloperoxidase, and the altered production seems to normalize in case of remission of the disease (109).

In CLL, an altered NK cell activity with transcriptional decrease of numerous cytotoxic signaling and decreased stimulating receptor expression has been reported. The NK-cell alteration appears to be of clinical importance, as greater NK-cell amounts is described in subjects with initial disease good prognosis (mutated IGHV genes), and better NK cytolytic ability is described in subjects with monoclonal B-lymphocytosis (110).

CLL cells generate numerous depressing signals that exert several negative actions on cell-related immunity. For instance, expression of CD200 stimulates differentiation of CD4+ T cells into T regulatory cells, which present CTLA-4, CD270 and PD-L1; these molecules, interacting with their receptors, are able to decrease T-cell growth and stimulation (110).

Gene expression profiling of activated CD4+ cells demonstrated a different modification of T helper function, with a decrease of T-cell receptor signaling and a decrease of cytokine production. This alteration was extremely pronounced in initial CLL but was also demonstrable in other lymphoproliferative diseases, such as follicular lymphoma and extranodal marginal zone lymphoma (111).

Moreover, in CLL, clonal B cells block the activity of normal T lymphocytes *via* the alteration of the establishment of the immunologic synapse in a contact-dependent way (112–114), and being leukemic cells, such depressing cell contacts can be permanent and ubiquitous (115).

A study demonstrated a modification of genes implicated in cell differentiation and cytoskeletal formation in CD4+ T cells, and cytoskeletal formation, vesicle transferring, and cytotoxicity activity in CD8+ T cells. Similar changes in cytoskeletal structure could be produced in healthy allogeneic T cells by co-culturing them with CLL cells. Moreover, modifications in cytoskeletal genes cause a functional alteration in actin polymerization. The result is that T cells from CLL subjects show defective immunologic synapse formation with APCs (107).

Nevertheless, other immunologic alterations are verifiable in these subjects generally due to the same disease or to the treatment (116–122).

Tinhofer et al. reported the existence of CD95 ligand on the cellular membrane of CLL cells, with the molecule being a ligand for the death receptor CD95 (123, 124). Furthermore, an augmented presence of surface CD95 on CLL subjects’ CD4+ T cells was demonstrated. These findings suggest that CLL cells could quickly reduce the helper T cell activity *via* this system, which would determine the humoral immunodeficiency in CLL patients (124).

Finally, Sampalo et al. demonstrated the negative action of CLL cells on the plasmatic cell, and the depressing action on the generation of normal immunoglobulin by plasmatic cells was correlated to increased amounts of CLL cells (125).

### Prophylaxis and Therapy of Secondary Immunodeficiency in Patients With Chronic Lymphocytic Leukemia. Effects of Chemotherapy on the Onset Of Infections

The simple treatment of CLL does not reestablish a normal immune response and guidelines do not suggest the presence of SID as a motivation for undergoing therapy (126).

Prompt identification of CLL subjects vulnerable to infections and prophylactic dispensation of correct antibiotics is the first approach for the treatment of an antibody insufficiency or SID in patients with CLL (120, 121). In subjects suffering with bronchiectasis, nebulized or low oral dosages of nebulized antibiotics, such as azithromycin, can decrease the occurrence of frequent infections.

The existence of a correlation between the concentrations of a specific class of antibody and the nature of pathogen producing the infection is a debatable issue. However, several authors believe that a reduced production of IgG is often accompanying an infection with streptococcus or haemophilus (107). Instead, IgA reduction is correlated with an augmented occurrence of upper respiratory system infections.

Significant reduction of normal immunoglobulin has been identified to be one of the elements accountable for the augmented vulnerability of CLL subjects to infection; this is the motivation for the use of IG in these subjects (127, 128).

Immunoglobulin replacement therapy (IgRT) may be dispensed intravenously (IV) or subcutaneously (SC). In SID, the effectiveness and safety of SCIG have been reported in numerous subjects with lymphoproliferative diseases and hypogammaglobulinemia (128, 129).

SCIG might present numerous benefits over IVIG as SCIG has been correlated to an enhancement in the quality of life perceived by the patient (130). Moreover, pharmacokinetic characteristics seem greater, as SCIG administration cause higher and more constant IgG concentrations, offering CLL subjects with a more stable defense against infections (131).

In a European consensus declaration, the quantification of serum Ig levels and the concentrations of specific antibodies after vaccination was established as a suitable method for CLL SID subject selection to employ IG (132). However, while in Canada, IVIG and SCIG preparations are commonly employed in SID patients (133, 134), in the EU the accepted indications for IVIG in SID have been expanded from CLL subjects undergoing to allogeneic hematopoietic stem cell transplantation, to subjects who present grave or frequent bacterial infections, unsuccessful antibiotic therapy and serum IgG concentration of <4 g/L (135–138).

Nevertheless, a 14-year retrospective report performed on a big number of CLL subjects established that hypogammaglobulinemia does not seem to modify overall survival (139) and, based on the findings of the first controlled trial on a wide cohort of CLL subjects, IV IgRT was not cost-effective (140). Consequently, the EMA is presently revising its guidelines on the employ of IVIG to evaluate secondary immunoglobulin deficit (141). Their suggested dosage is 0.2–0.4 g/kg every 3 to 4 weeks. However, there is supplemental evidence that SCIG provides efficacious protection in subjects with secondary antibody deficiency (142).

Although IgRT is usually well tolerated in subjects with hematological malignancies, IgRT can on uncommon cases provoke to adverse effects such as hypersensitivity, kidney failure, thromboembolism and hemolysis. Thus, IVIg administration should be carefully examined, especially in subjects with risk factors. Sufficient hydration is crucial. Moreover, when commencing IgRT in subjects with hematological malignancies, the dosage should be weight-based. In obese subjects, IgRT dose should be established on an ideal or adjusted body weight. In any case, SCIg administration might present a lower risk of systemic adverse events. For this reason, in subjects undergoing therapy for hematological malignancies who are about to begin IgRT, both SCIg and IVIg should be evaluated. Patients should be implicated in the decision on the best route of administration considering their preference. Finally, discontinuation should be considered only after at least 6 months without infections and if there is evidence of immunological recovery. Infection rates should be closely evaluated and IgG levels should be tested during routine patient visits.

**Figure 3:**
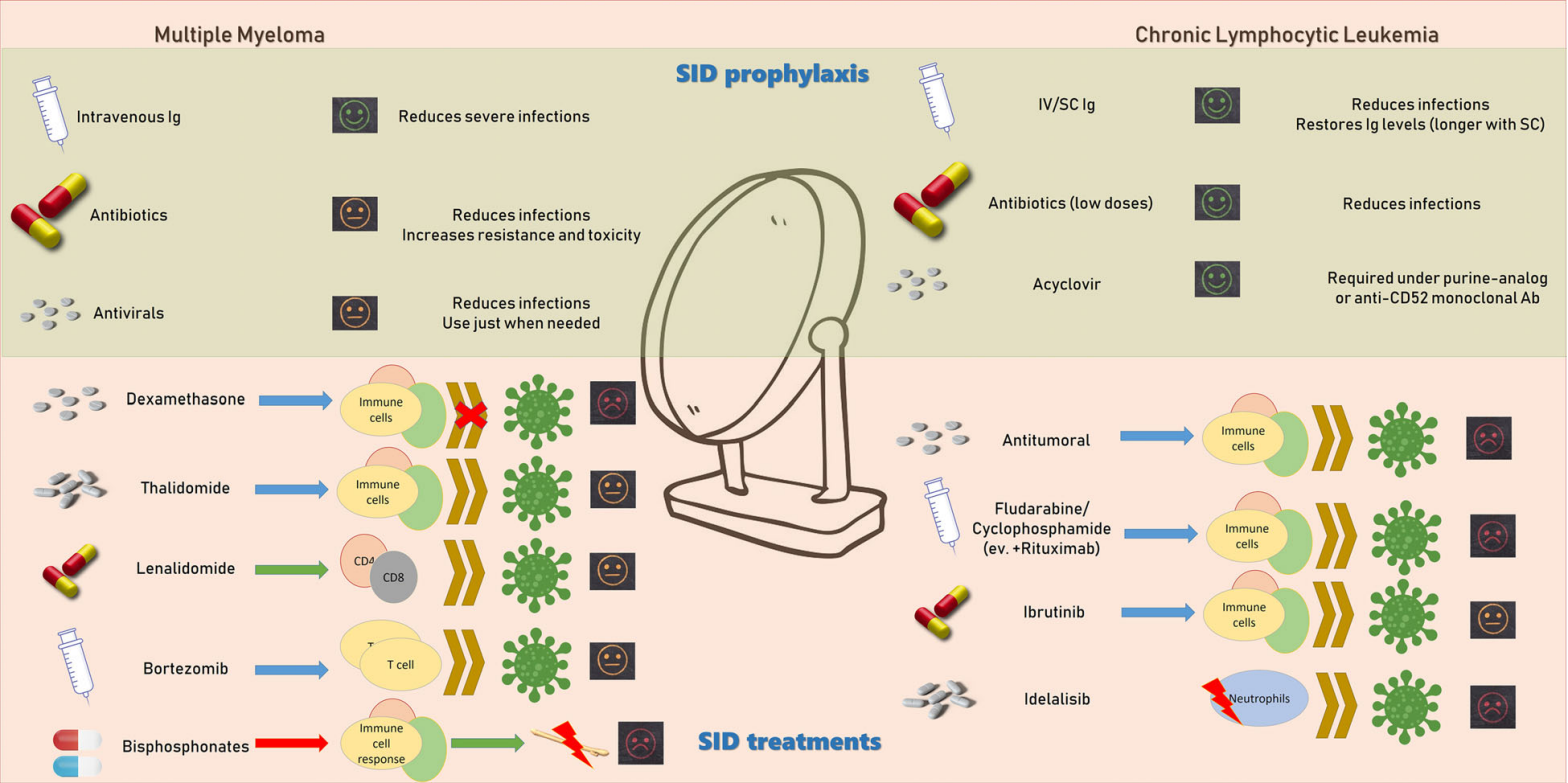
Possible immunological targets of drugs used in the prophylaxis and treatment of secondary immunodeficiency of patients with multiple myeloma and chronic lymphocytic leukemia. Action of antineoplastic drugs on immunological effectors.

Particular attention should be paid to patients with CLL undergoing therapeutic treatment (**Figure 3**).

An adequate prophylaxis is suggested for subjects getting purine- analogue or an anti-CD52 monoclonal antibody, such as alemtuzumab, both during and after therapy. Moreover, a prophylaxis with acyclovir for herpes virus and a *Pneumocystis jirovecii* prophylaxis employing sulfamethoxazole/trimethoprim should be used (Grade of evidence IV, B) (143, 144).

The augmented utilization of new B‐cell targeted treatments capable of modifying the function, the differentiation and programmed cell death of B cells, and the use of CD19‐targeted chimeric antigen receptor T cells (CAR T) in lymphoproliferative diseases have augmented the occurrence of SID in hematological malignancies (145, 146).

Specific antitumor drugs, as combination chemotherapy, can also increase the risk of some viral infections (91, 101). It has been proposed that these therapeutical protocols synergistically reduce myeloid cells, causing increased immunosuppression and a resulting augmented risk of infection (147, 148). After combination chemotherapy, a higher percentage of grave or uncommon infections have been reported than in subjects treated with fludarabine or cyclophosphamide alone. However, a greater infection-related mortality has not been described by the existing literature. The employment of rituximab, an anti-CD20 monoclonal antibody, in combination treatment with fludarabine and cyclophosphamide (FCR) augments the occurrence of different forms of infections (149). FCR protocols cause deep myelosuppression and a relevant incidence of infections, particularly in older people. Anti-CD20 monoclonal antibodies have been correlated with cytomegalovirus infection or reactivation of hepatitis B and polyomavirus JC infections (150, 151). Recurrence of herpes viruses have also been reported after administration of purine analogs and alemtuzumab (152).

As far the mechanisms underlying the immunosuppressive effects, rituximab provokes a fast reduction of CD20- presenting mature and immature B-cells, which persist at very low concentrations for about 9 months (153). Moreover, rituximab can cause a condition of immunosuppression also *via* the occurrence of a deep neutropenia and a reduction of immunoglobulin levels (154–156). Furthermore, studies also propose that rituximab augments the risk of progressive multifocal leukoencephalopathy (157–159), while an altered humoral immune response with inadequate B-cell growth to simple haptens and recall antigen challenge propose an unfavorable action on memory B-cells (160–162).

Of particular interest is the comparison between the ability of traditional drugs to induce a state of immunosuppression compared to new drugs such as Bruton’s kinase inhibitors, phosphatidylinositol 3-kinase inhibitors or B-cl2 inhibitors.

Numerous studies have evaluated the impact of new drugs for the treatment of CLL on the onset of infections. In a multicenter clinical trial valuing ibrutinib (a Bruton’s tyrosine kinase inhibitor) *versus* an anti-CD20 monoclonal antibody such as ofatumumab in refractory CLL, critical infections of respiratory tract, and urinary tract were analogous in the two groups (24 *versus* 22% of subjects), although CLL subjects on ibrutinib treatment had lengthier treatment exposure (163). The percentage of critical infections was inferior when ibrutinib was administered as first-line therapy (164) (Burger et al., 2015). In the clinical trial with idelalisib (a phosphatidylinositol 3-kinase inhibitor) for refractory CLL, different infections were described such as pneumonia (6%) and febrile neutropenia (5%) (165); a diverse study performed with idelalisib for diverse lymphoproliferative disorders confirmed the occurrence of grave neutropenia (27%) and pneumonia (7%) (166), while a clinical trial on CLL subjects stated 18% occurrence of pneumonia (167). In a phase II clinical trial in refractory CLL, Bcl-2 antagonists, such as venetoclax, have demonstrated an incidence of pneumonia in 6% and febrile neutropenia in 5% of subjects (168).

Regarding the characteristics of immunosuppression induced by new drugs, a study performed on patients submitted to a long-lasting treatment with ibrutinib propose a reduction in the infection occurrence during therapy (169). Two different explanations were suggested. The reduction of the interleukin-correlated T-cell kinase, which can stimulate T-helper cell type 1 CD4 T-cell growth, provoking a decrease of infections, or a restoration of the humoral immunity with an increased production of immunoglobulin A. However, the real reason of an improvement in immunoglobulin function after prolonged treatment remains unidentified (170).

However, it is clear that such new drugs have a lower impact on the immune system of patients with CLL and could guarantee a better safety profile, better adherence to therapy and ultimately a better outcome.

Finally, special consideration was given to the occurrence of neutropenia after therapy with these molecules. The employ of granulocyte-colony stimulating factor (G-CSF) in subjects assuming ibrutinib is infrequent. However, in CLL subjects developing grave neutropenia (ANC < 1.0×109/L), G-CSF has been administered in clinical trials and no side effects were described.

Idelalisib has been correlated to a greater occurrence of grave neutropenia with respect to ibrutinib. The median time to onset of grade ≥ 3 neutropenia was 1.4 months (171), and G-CSF has been administered in 25% of subjects (165).

A phase 1 clinical trial of venetoclax in relapsed/refractory CLL described a 17% of grave infections. The percentage of grade 3/4 neutropenia was 41%, although the percentage of febrile neutropenia was only 6% (172). Analogously in a phase 1b study of venetoclax plus rituximab in relapsed/refractory CLL, the percentage of grave infections was 16% while the percentage of grade 3/4 neutropenia was 53% (173). A phase 2 clinical trial of venetoclax in relapsed/refractory CLL with bad prognosis (del(17p)) described a percentage of grade 3/4 neutropenia of 40%, and febrile neutropenia of 5% (168).

Finally, the number of preceding treatments seem relevant for viral and fungal infections (174).

## Effects of Vaccination in Patients With Secondary Immunodeficiency

Safeguarding SID subjects against vaccine-avoidable infections is a frequently ignored field of study (175). However, SID is an obstacle to efficacious vaccination in patients with tumors and chronic infection.

There are essentially two difficulties preventing effective vaccination therapy. The first is the problem that the live vaccine, even if attenuated, could provoke a grave infection. Second, the response to the vaccine in such patients might be inadequate and, subsequently, non-protective (176).

These considerations are particularly valid in patients with hematological malignancies undergoing chemotherapy treatment. An approach with vaccination before the onset of SID would guarantee security and would assure an optimal immune response.

Elements able to condition the effectiveness of vaccination before the onset of SID include the type of hematological malignancy, the vaccine dosage, and the type of chemotherapy treatment given before vaccination.

Prognostic stratification also appears to be a factor capable of influencing the response to vaccination. In a report, after the end of chemotherapy, patients in the low-risk-group demonstrated more often satisfactory antibody concentration against tetanus and diphtheria than patients of the high-risk-group. However, antibodies against tetanus appeared in about 50% of all subjects, and subsequently, re-vaccination antibodies against tetanus were demonstrated in approximately all subjects whereas for diphtheria, this occurred only in some patients. Remarkably, subjects without adequate antibody concentrations displayed a sufficient cellular immune response (177).

Influenza and Pneumococcal vaccination are suggested in early-phase CLL (178), as the vaccination against *Neisseria meningitides*, *Streptococcus pneumonia*, *Haemophilus influenzae*, and other encapsuled pathogen. Vaccination should be performed at least 2 weeks before the beginning of chemotherapy or 3 months after the end of chemotherapy, or 6-12 months after the employ of an anti-B-cell antibody treatment (179). This is mandatory if patients are functionally asplenic or have to be submitted to a splenectomy (180) (Rubin et al., 2013).

Although there are no controlled trials demonstrating that the use of vaccinations may modify infection percentages or prognosis from infectious diseases in CLL, standard vaccinations should be performed in these subjects before the starting of a therapy (179). All live vaccines should be avoided, while conjugate vaccines have proven to be extremely immunogenic and should be suggested in CLL subjects (181).

Lower responses to vaccination in CLL subjects with respect to control subjects have been demonstrated against diverse antigens. Specific antibody generation to polysaccharide antigens, which is expression of a T-cell- independent response, such as classical 23-valent pneumococcal vaccine, is significantly altered in CLL subjects (182). Flawed response to different protein antigens, such as tetanus toxoid is also evident (183, 184).

However, evaluation of vaccine response is difficult and requires special attention. For the analysis of primary responses, an augment higher than treble after 4 weeks with respect to pre-vaccination concentrations is judged adequate (185, 186).

Hepatitis B vaccine is also suggested for CLL subjects who are devoid of antibodies against HBsAg, and a combination of hepatitis A and B vaccine should be evaluated in endemic areas (187).

However, the bad success of hepatitis B vaccination in the greater part of subjects with indolent lymphomas suggests an altered immune response against this type of infection (188). Failure to response to vaccination may be provoked by a T-cell alteration. A greater rate of ageing CD8+ CD28- T cells presupposes failure to vaccination (189, 190). A decreased naive T-cell set might diminish the amount of T cells presenting a T cell receptor (TCR) that identifies an extraneous antigen, such as HBs. Finally, transcriptional reduction of TCR signaling may also alter T-cell sensitivity to antigenic stimulation.

A reestablishment of a normal immune response could not only allow successful vaccination of CLL subjects but could theoretically enhance prognosis (191, 192).

As far MM subjects, in the CAPiTA clinical trial of 84,496 patients (NCT00744263), a vaccine effectiveness of 45,56% for the 13-valent pneumococcal conjugate vaccine was reported. Vaccination could also help in the prevention of other frequent infections in MM subjects, such as meningitis, influenza, and varicella (193).

Finally, another important obstacle could be due to an altered activity of antigen presenting cells, which are altered in amount and activity (194). In order to bypass this difficulty, the employ of immune adjuvants has been proposed (195).

Despite what has been said about the undoubted opportunity for vaccination of patients with MM and CLL, there are disadvantages and advantages that must be carefully weighed in each patient. Indisputable advantages are prevention of high mortality diseases and complications, the possibility to reduce the use of antibiotics and antimicrobial, resistance development, and finally, the possibility of a herd immunity. However, it must also be considered that inactivated vaccines may be less effective and live-attenuated vaccines have increased risk for adverse reactions. For these reasons, an expert in vaccination should be included for the management of MM and CLL patients to choose the timing, dosage and the need for a possible repeat vaccination in this specific population.

Particular considerations should be done for subjects undergoing autologous bone marrow transplantation (ABMT). In 1995 the Infectious Diseases Working Party of the European Group for Bone Marrow Transplant presented endorsements for re-immunization in subjects who had submitted to allogeneic or ABMT (196) and these recommendations were successively reworked (196), while the Centers for Disease Control and Prevention formulated their proposals (197). Agreeing to these suggestions, a complete vaccination schedule against tetanus, diphtheria, and poliomyelitis is intensely suggested post autologous transplantation for all subjects with hematological malignancies. Moreover, vaccinations against hepatitis B, Haemophilus influenza type B, and influenza are suggested in specific subsets of subjects, while a vaccination therapy aimed at prevention of infections by mumps, pneumococci, rubella, or measles, should be programmed on the characteristics of the individual patient.

A report evaluated if the modifications of immune response described after ABMT would continue in the years after the procedure has been performed. Subjects with lymphoma submitted to ABMT were enrolled. Median time from ABMT was 5 years. Immunophenotyping demonstrated an augment in the amount of B cells, and a reduction in T cells. Moreover, a minor amount of CD4+ T cells caused a diminished CD4/CD8 ratios. The rate of patients presenting CD4+ T cells expressing the ‘naive’ phenotype CD45RA was 19.5% *vs* 38% in controls. In contrast, the rate of patients expressing the memory phenotype CD45RO was greater in the ABMT group (76% *vs* 54%). After stimulation, greater parts of CD3+CD4+ cells in lymphoma subjects generated IFN-gamma (32% *vs* 16%) or IL-4 (7% *vs* 1%) with respect to healthy subjects. A prevalent Th2 response was described in patients (198).

A different study evaluated the delayed consequences of ABMT on the immune response with regard to vaccines. Vaccination was administered according to EBMT suggestions. The study involved subjects with lymphomas in complete response 4–10 years after ABMT, and a control group. The findings demonstrated that before ABMT the rate of subjects with protective immune response against diphtheria, poliomyelitis, and tetanus was analogous to that of healthy subjects. 4–10 years after ABMT, the percentage of subjects with adequate immune response against diphtheria and poliomyelitis was decreased, while all lymphoma subjects conserved protection against tetanus (199).

In 2017, the U.S. FDA approved the adjuvanted zoster vaccine. Phase 1/2a trials performed on SID patients comprising ABMT receivers and HIV patients, have established that this vaccine is efficacious and secure with no events of varicella zoster infection (200). Currently, the vaccine is suggested in more than 50 years old patients with hematologic malignancies.

Finally, Rapoport et al. accomplished a phase 1/2 trial in patients with a reduced number of lymphocytes after high-dose chemotherapy and ABMT for MM (201). Early post-transplant administration of *in vivo* vaccine primed, and *ex vivo* co-stimulated autologous T cells ensued by post-transplant immunizations reduced the grave SID due to high-dose chemotherapy.

## Onset of Infectious and Non-Infectious Complications in Hematological Patients With Secondary Immunodeficiency

With respect to healthy controls, patients with hematological malignancies undergo several chronic complications correlated to SID, comprising critical, or fatal situations such as endocrine alterations, cardiac and pulmonary diseases, and successive tumors (202). Host risk elements comprise age, and pathological conditions present, while disease‐correlated components are motionlessness and the need to control a painful syndrome. Indeed, comorbidities augment with age, and there is an age‐correlated deterioration in functional resources in different systems comprising immune response. Moreover, gerontological alterations, cognitive decline, and community separation all participate to the possibility of complications (203).

Furthermore, there is a bidirectional relationship between non-infectious complications and the onset of infections in patients with hematological malignancies. For instance, the employ of morphine or other pain relievers for bone pain can alter respiratory function, while renal failure is also a risk element for infectious diseases in MM patients, with about 20% of MM subjects displaying an altered renal function. Amyloidosis, and heart failure can arise as consequence of MM, and this is also linked to an augmented risk of infections.

As reported above, infections can cause a state of immunodeficiency, capable of affecting other complications. For instance, B CLL subjects may present a second neoplasm with an occurrence greater with respect to normal subjects (204). In a study, substantial augments were reported for Kaposi sarcoma, lung cancer, melanoma, and laryngeal carcinoma (205). As potential mechanism able to augment the risk of second malignancies in CLL patients, authors propose the immunodeficiency correlated to disease. The effects of SID associated with infections is unclear, but it cannot be excluded or underestimated (205, 206).

The data relating to specific, rare types of cancer appear interesting. Merkel cell carcinoma (MCC) is a neuroendocrine skin tumor with a great tendency for relapse and diffusion. Risk elements for MCC comprise UV exposure, older age, and immunosuppression (207–209). The Merkel cell polyomavirus (MCPyV) is accompanying with MCC in ∼60% to 80% of subjects, proposing an essential effect of the infection and the SID in the onset of this tumor (210). An immunosuppressed condition may stimulate the different phases of MCPyV incorporation, mutagenesis, and carcinogenesis. Indeed, immunosuppressed subjects are over-represented among MCC subjects with respect to normal subjects. CLL has been correlated with augmented risk of MCC (211).

Changes of immune responses may also modify the response to treatments comprising radiation. Local relapse after palliative radiotherapy is greater among MCC immunosuppressed subjects with respect to immunocompetent patients (35% *versus* 9%). Analogous results were found after standard doses of curative-intent radiotherapy on progression free survival among MCC subjects with immunosuppression (212, 213).

Thrombosis is the second most usual cause for death in tumor-affected subjects, and this is due to several factors. In fact, tumor subjects generally present a hypercoagulable state without a clear origin.

Also in this case, there is a bidirectional relationship between non-infectious complications and the onset of infections in patients with hematological malignancies. Although a state of immunodeficiency is generally characterized by a reduced production of NET, as reported above, a dysregulated activation of innate immunity can determine an altered NET production. Recently, NETs were found to cause a hypercoagulable condition, and stimulate the onset of thrombosis (214, 215). Studies performed both in experimental models and in clinical studies have demonstrated that circulating concentrations of NETs are augmented in several forms of tumors (216–218) and may participate to tumor-correlated thrombosis (219). For example, Podaza et al. have reported that neutrophils extracted from CLL subjects presented an augmented ability to discharge NETs, and plasma from CLL subjects is able to instruct neutrophils from normal subjects to produce greater levels of NETs after ex vivo stimulation (218). Likewise, in animal experimental model of chronic myeloid leukemia, neutrophils from leukemic animals were more inclined to produce NETs after stimulation by platelet-activating factor with respect to controls (220). Moreover, NETosis is correlated with lung thrombosis in an experimental model of breast carcinoma, while administration of a low dose of lipopolysaccharide in tumor-bearing animals augments plasma NET biomarkers and causes a prothrombotic condition (220)

Pulmonary emboli usually happen within the first 21 days after transplantation and can be accompanied by fever. Ramsey et al. reported that bacterial infections had occurred in nearly all patients who had had a premortem diagnosis of pulmonary emboli. Opportunistic infections almost always happen within six months, when immunosuppression is highest (221). Pulmonary emboli should be uncommon beyond this time.

A different pathogenetic mechanism is probably the cause of the onset of the hepatic sinusoidal obstruction syndrome (SOS), an obliterative venulitis of the terminal hepatic venules, which is accompanied by an elevated risk of death. SOS, also known as veno-occlusive disease (VOD), happens as an effect of chemotherapy prior to hematopoietic stem cell transplant. An essential pathogenic mechanism is toxic damage of hepatic sinusoidal endothelial cells, with alteration and obstruction of terminal hepatic venules. Causal elements are augmented intrahepatic presence of matrix metalloproteinases and vascular endothelial growth factor, stimulation of clotting factors, sinusoidal endothelial cells glutathione reduction, and nitric oxide diminution. However, a correlation of SOS with SID has been reported (222).

Interesting studies have been conducted on the occurrence of ocular changes in SID patients. Several systemic pathologies as leukemia and anemia, thrombocytopenic purpura, systemic lupus erythematous, diabetes, HIV/AIDS, and hypertension, are recognized to be able to cause retinal lesions (223, 224).

The association of retinal changes with bacterial infections has long been identified (225, 226). Similarly, occurrence of candidal endophthalmitis was also recognized. Two different forms of retinal damage have been reported in the two types of infections: septic emboli, which may cause endogenous or metastatic endophthalmitis, and nonspecific retinal alterations, comprising retinal hemorrhages, cotton wool and Roth spots (227). Moreover, candidal endophthalmitis has been divided into two other types established on the size of the alterations (228). If the infectious event is concentrated within the chorioretinal layers, the name chorioretinitis has been employed. When, in addition to the chorioretinal participation, there is a vitreal abscess, then the expression candidal endophthalmitis is used (229).

Several complications involving the respiratory tract are only in part attributable to infections and in any case find the primum movens in a state of profound immunodeficiency. Opportunistic fungal infections, especially Aspergillus, are a usual reason of morbidity and death in immunocompromised patients and can appear with pneumonia and central airway blockade (230–232). Cases of bronchomediastinal fistula have been described in CLL subjects (233), and bronchomediastinal fistula provoked by endobronchial Aspergilloma may provoke death. Forceful therapy with antifungals and bronchoscopic procedures are mandatory.

Similarly, in studies on pulmonary alterations in immunocompromised patients, the expression interstitial pneumonitis has been employed to designate diverse conditions. Some researchers comprise all parenchymal alterations, while others employ this expression. Finally, other authors employ it for idiopathic pneumonitis, referring to those patients in which no precise diagnosis is present. Interstitial pneumonitis in the subjects with hematologic malignancies who experienced bone marrow transplantation has a diverse meaning. It can be due to cytomegalovirus infection or may be idiopathic. However, infrequently, it may be due to *Pneumocystis carinii*, herpes simplex, and it is correlated to a high mortality (more than 50%) when the subject presents severe graft-versus-host disease (233, 234). Introduction into clinical practice of prophylactic employ of trimethoprim sulfamethoxazole in these subjects reduced the onset of *P. carinii* infections (235).

Clinical symptoms of interstitial pneumonitis after allogenic BMT are characterized by the presence of fever, rattles, tachypnea, gasp, reduced Pa02, and radiologic evidence of an interstitial process. Generally, interstitial pneumonitis starts two months after transplantation and happens in 20 to 30% of recipients. The development of interstitial pneumonitis was an independent variable shown to significantly impact on TRM and OS (236).

Aspiration pneumonia, which generally happens in the debilitated subjects, may dispose the SID patient to secondary bacterial infections, comprising anaerobic infections and their complications. The adult respiratory distress syndrome, happens acutely and simulates other acute complications.

Rather frequent causes of pleural effusion in the SID patients are bacterial pneumonia, comprising that provoked by *Legionella*, while unusual causes are *Nocardia*, and rare are *Pneumocystis, C*ytomegalovirus, Herpes virus, and invasive fungal disease (235).

### Orofacial Manifestation

Orofacial expressions of hematological malignancies and SID are frequent in leukemic patients (237) (Adeyemo et al., 2011). Gingival enlargement with limited or complete cover-up of the crowns of the teeth is a usual finding, especially in acute monocytic leukemia, and it is due to the total permeation of the gingiva by clonal cells. The augmented predisposition to oral bleeding prejudices an adequate oral hygiene and provokes gathering of microbial plaque which operates as an inflammatory incentive for an overstated reaction to plaque with consequent formation of connective tissue hyperplasia of the gingival and augmented periodontal damage (238–241).

Neutropenic mucosal ulcerations are reported in nearly half of leukemic subjects and this complication happens when the number of neutrophils falls below 1000 neutrophils/mm (242). Augmented vulnerability to infections and appearance of pericoronitis, or periapical and periodontal aching inflammations are common in SID patients (243–245) as necrotizing ulcerative gingivitis, and hairy leukoplakia (246). Palatal necrosis and necrotic lesions of the palate may be found in subjects with acute leukemia (247).

In leukemic patients, the immunosuppressive actions determined by the treatment, especially steroid therapy, can provoke the onset of oral mucositis, which can happen without a viral participation, as thinning of the mucosa and the presence of bone marrow inhibition which permit the onset of other opportunistic infections able to damage mucosa. This may be avoided by scrupulous buccal cleanliness and administration of topical antimicrobial drugs (248).

MM subjects have a deep immunosuppression; thus, several forms of buccal alterations may be recognized in this type of patients. These comprise oral hairy leukoplakia (249, 250), while amyloid accumulation in the tongue can also provoke the onset of macroglossia with atrophic tongue or translucent papules on the buccal mucosa or the tongue (251).

## SID and Cancer Risk

As highlighted, the relationships between neoplasms and infections are bidirectional. While it is true that tumors inducing immunosuppression are capable of causing the appearance of infections, it is also true that many infections have an oncogenic capacity. Chronic infection is one of the main reasons of tumor, and there are numerous pathogenetic moments to explain infection-correlated oncogenesis, and microorganisms can provoke the onset of tumors through the determination of important and chronic inflammatory states, the implementation of an intrinsic oncogenic potential, and above all the onset of a severe immunodeficiency capable of conditioning escape from host immunity (252).

SID subjects are at augmented risk of acquiring lymphoproliferative neoplasms, as indeed found in patients with primary immunodeficiency (253).

In MM patients, microorganisms presenting pathogen-associated molecular patterns can stimulate toll-like receptors on MM cells, hasten MM cell proliferation and inhibit chemotherapy-provoked programmed cell death (254–256). Furthermore, by stimulating the delivery of immune mediators, infections can speed up tumor advancement (257, 258). For this, by rapidly eliminating the pathogens and changing the pro-inflammatory immune microenvironment, drugs with anti-infective activity can have both anti-tumor and anti-infection capacities.

Based on what has been said, it is evident that a careful evaluation of the state of immunocompetence is necessary in all patients with hematological malignancies. To assess the effect of immunological changes and to control subject suffering from hematological malignancies, a careful clinical history of infections, an evaluation of serum immunoglobulins (259) and a quantification of lymphocyte subsets, comprising both CD4, CD8 T cells and B cells are suggested at the diagnosis and after chemotherapy. Neutrophil assessment should be also frequently controlled (260).

A study has underlined the extreme relevance of regular routine immunological appraisal for secondary specific antibody insufficiency to protein immunization in CLL patients as an approach for identifying the most susceptible subjects to infections. These analyses should be performed every 6–12 months and always after the appearance of relevant infections, and after a chemotherapy (91).

## Future Perspectives

A promising field of study appears to be that of drug repositioning, with the possibility that drugs employed for the therapy of infectious diseases may be advantageous in the treatment of hematological neoplasms, especially in immunosuppressed patients (261). It was established *in vitro* that the anti-infective chloramphenicol could stimulate MM cell death in a dose- and time-dependent way (262). Contemporarily affecting two essential intracellular protein degradation mechanisms of the ubiquitin-proteasome and the autophagylysosome system, chloramphenicol causes endoplasmic reticulum stress related apoptosis and the production of the pro-apoptotic transcription factor CADD153 able to induce programmed cell death in MM cell lines (263). Further, thalidomide and clarithromycin were reported to synergistically decrease IL-6 and TNF-α production possibly through ERK1/2 and AKT block (264).

A different area of study could be constituted by the serial evaluation over time of the immunological alterations that characterize conditions such as MGUS and MBL. It is in fact known that patients with MBL, a precursor to CLL, show defective synapse formation between B cells and T cells which worsens with MBL’s progression to CLL. A careful study of these patients could uncover the immunological mechanisms that favor the progression of these conditions towards an overt disease and could suggest new approaches to prophylaxis and therapy for both hematological neoplasms and infectious complications.

Finally, a different issue that should certainly be studied is that of vaccination of patients with hemopathies due to the difficulties it manifests and the possibilities it offers, and the implications inherent in vaccination practice in patients with hematological diseases emerge strongly in the current pandemic period. Patients with hematological malignancies have an augmented risk for critical COVID-19 disease and mortality. Herishanu et al. evaluated immune response to the BNT162b2 messenger RNA COVID-19 vaccine in CLL subjects. A comparison between patients and healthy controls demonstrated a significantly diminished response rate among patients. The response was greater in CLL subjects who attained clinical remission, followed by treatment-naive patients and only 16.0% in subjects under treatment at the time of vaccination. In CLL subjects treated with Bruton’s tyrosine kinase inhibitors or venetoclax ± anti-CD20 antibody, response rates were significantly low. None of the CLL subjects treated with anti-CD20 antibodies <12 months before vaccination responded (265). All this confirms the difficulty of vaccination in patients with hematological malignancies.

A possible useful approach could be to vaccinate subjects at Monoclonal Gammopathy of Undetermined Significance and Monoclonal B Lymphocytosis stages (for MM and CLL, respectively) or at initial phase of B-cell malignancy diagnosis, when a better antibody response could be obtained with respect to more advanced stages (260).

Moreover, many of the difficulties encountered in vaccinating SID subjects are like those encountered in implementing an adequate vaccination against tumors. Nevertheless, vaccino-therapy aimed at the treatment of tumors has great prospects in treating hematological malignancies and may offer a complementary treatment modality to treat these diseases with respect to traditional chemotherapy. New approaches comprise genetic manipulation of autologous T cells with receptors for tumor-specific epitopes, administration of vaccine-primed and *ex vivo* costimulated autologous T cells after high dosages of immunosuppressive drugs such as melphalan, infusion of dendritic cell/plasma cell fusions and employ of expanded marrow-infiltrating lymphocytes. The mission in the future is to assess these strategies in apposite clinical groups of patients, and to combine them with schemes to overwhelm immunoparesis as a method to generate a strong immune response (266, 267).

## Conclusions

In conclusion, the mechanisms underlying the appearance of SID in patients with hematological malignancies are different and are correlated to the neoplasms and the same treatments. The multiplication in therapeutic opportunities for these diseases makes it of utmost importance to gain a better understanding of the effects of new therapies on the various components of the immune system and the prevention of infectious complications. Immunotherapies are revolutionizing the treatment of hematological malignancies. Moreover, by suppressing the pro-inflammatory microenvironment and pathogens, prophylactic and therapeutic antibiotics represent anti-tumor and anti-infection properties. Combined therapies integrated with optimized modern clinical management will continue to deliver better anticipations for patients in years to come. Hopefully, the collaborative efforts from hematologists, infectious disease specialists, pathologists, pharmacologists will ensure the best management of these patients.

## Author Contributions

Conceptualization, SG and AA. Methodology, CM AT and GP. Software, AT. Formal analysis, AT, CM, GP. Data curation, GP and AT. Writing—original draft preparation, AA. Writing—review and editing, AA and SG. Supervision, AA and SG. All authors have read and agreed to the published version of the manuscript.

## Conflict of Interest

The authors declare that the research was conducted in the absence of any commercial or financial relationships that could be construed as a potential conflict of interest.

## Publisher’s Note

All claims expressed in this article are solely those of the authors and do not necessarily represent those of their affiliated organizations, or those of the publisher, the editors and the reviewers. Any product that may be evaluated in this article, or claim that may be made by its manufacturer, is not guaranteed or endorsed by the publisher.

## References

[1] Ballo O Tarazzit I Stratmann J Reinheimer C Hogardt M Wichelhaus TA. Colonization With Multidrug Resistant Organisms Determines the Clinical Course of Patients With Acute Myeloid Leukemia Undergoing Intensive Induction Chemotherapy. PloS One (2019) 14(1):e0210991. doi: 10.1371/journal.pone.0210991 30673776 PMC6343922

[2] Šedivá A Milota T Litzman J Quinti I Meyts I Burns S. Medical Algorithm: Diagnosis and Management of Antibody Immunodeficiencies. Allergy (2021) 26. doi: 10.1111/all.14961 34037990

[3] Chinen J Shearer WT. Secondary Immunodeficiencies, Including HIV Infection. J Allergy Clin Immunol (2010) 125(2 Suppl.):S195–203. doi: 10.1016/j.jaci.2009.08.040 PMC6151868 20042227

[4] Truong NTH Gargett T Brown MP Ebert LM. Effects of Chemotherapy Agents on Circulating Leukocyte Populations: Potential Implications for the Success of CAR-T Cell Therapies. Cancers (Basel) (2021) 13(9):2225. doi: 10.3390/cancers13092225 34066414 PMC8124952

[5] Sánchez-Ramón S Bermúdez A González-Granado LI Rodríguez-Gallego C Sastre A Soler-Palacín P. ID-Signal Onco-Haematology Group. Primary and Secondary Immunodeficiency Diseases in Oncohaematology: Warning Signs, Diagnosis, and Management. Front Immunol (2019) 26:586. doi: 10.3389/fimmu.2019.00586 PMC6448689 30984175

[6] Kyle RA Rajkumar SV. Multiple Myeloma. N Engl J Med (2004) 351:1860–73. doi: 10.1056/NEJMra041875 15509819

[7] Allegra A Innao V Allegra AG Ettari R Pugliese M Pulvirenti N. Role of the Microbiota in Hematologic Malignancies. Neth J Med (2019) 77(2):67–80. 30895929

[8] Allegra A Speciale A Molonia MS Guglielmo L Musolino C Ferlazzo G. Curcumin Ameliorates the *In Vitro* Efficacy of Carfilzomib in Human Multiple Myeloma U266 Cells Targeting P53 and NF-κb Pathways. Toxicol *In Vitro* (2018) 47:186–94. doi: 10.1016/j.tiv.2017.12.001 29223572

[9] Ettari R Zappalà M Grasso S Musolino C Innao V Allegra A. Immunoproteasome-Selective and non-Selective Inhibitors: A Promising Approach for the Treatment of Multiple Myeloma. Pharmacol Ther (2018) 182:176–92. doi: 10.1016/j.pharmthera.2017.09.001 28911826

[10] Nishida H. Rapid Progress in Immunotherapies for Multiple Myeloma: An Updated Comprehensive Review. Cancers (Basel) (2021) 13(11):2712. doi: 10.3390/cancers13112712 34072645 PMC8198014

[11] van de Donk NWCJ Usmani SZ Yong K. CAR T-Cell Therapy for Multiple Myeloma: State of the Art and Prospects. Lancet Haematol (2021) 8(6):e446–61. doi: 10.1016/S2352-3026(21)00057-0 34048683

[12] Abbasi S Roller J Abdallah AO Shune L McClune B Sborov D. Hospitalization at the End of Life in Patients With Multiple Myeloma. BMC Cancer (2021) 21(1):339. doi: 10.1186/s12885-021-08079-x 33789626 PMC8011131

[13] Holmstrom MO Gimsing P Abildgaard N Andersen NF Helleberg C Clausen NA. Causes of Early Death in Multiple Myeloma Patients Who are Ineligible for High-Dose Therapy With Hematopoietic Stem Cell Support: A Study Based on the Nationwide Danish Myeloma Database. Am J Hematol (2015) 90(4):E73–4. doi: 10.1002/ajh.23932 25561348

[14] Public Health England. Routes to Diagnosis 2006–2016 Workbook. Version 2.1b. Survival by Route and Survival Time—Overall (2019). Available at: https://data.healthdatainsight.org.uk/apps/routes_to_.

[15] Blimark C Holmberg E Mellqvist UH Landgren O Bjorkholm M Hultcrantz M. Multiple Myeloma and Infections: A Population-Based Study on 9253 Multiple Myeloma Patients. Haematologica (2015) 100:107–13. doi: 10.3324/haematol.2014.107714 PMC4281323 25344526

[16] Zahid MF Ali N Nasir M Baig MH Iftikhar M Bin Mahmood SU. Infections in Patients With Multiple Myeloma Treated With Conventional Chemotherapy: A Single-Center, 10-Year Experience in Pakistan. Hematol Transfus Cell Ther (2019) 41(4):292–7. doi: 10.1016/j.htct.2019.02.005 PMC6978542 31412989

[17] Valkovic T Gacic V Ivandic J Petrov B Dintinjana RD ED H. Infections in Hospitalised Patients With Multiple Myeloma: Main Characteristics and Risk Factors. Turk J Hematol (2015) 32:234–42. doi: 10.4274/tjh.2013.0173 PMC4563199 26376590

[18] Lee SK Sung PS Park SS Min CK Nam H Jang JW. Reactivation of Resolved Hepatitis B After Daratumumab for Multiple Myeloma. Clin Infect Dis (2021) 73(6):1372–5. doi: 10.1093/cid/ciab302 33846712

[19] Su TH Liu CJ Tseng TC Chou SW Liu CH Yang HC. Chronic Hepatitis B is Associated With an Increased Risk of B-Cell non-Hodgkin’s Lymphoma and Multiple Myeloma. Aliment Pharmacol Ther (2019) 49:589–98. doi: 10.1111/apt.15132 30681172

[20] Takeshita M Sakai H Okamura S Higaki K Oshiro Y Uike N. Prevalence of Hepatitis C Virus Infection in Cases of B-Cell Lymphoma in Japan. Histopathology (2006) 48:189–98. doi: 10.1111/j.1365-2559.2005.02311.x 16405668

[21] Duberg AS Nordström M Törner A Reichard O Strauss R Janzon R. Non-Hodgkin’s Lymphoma and Other Nonhepatic Malignancies in Swedish Patients With Hepatitis C Virus Infection. Hepatology (2005) 41:652–9. doi: 10.1002/hep.20608 15723449

[22] Franceschi S Lise M Trépo C Berthillon P Chuang SC Nieters A. Infection With Hepatitis B and C Viruses and Risk of Lymphoid Malignancies in the European Prospective Investigation Into Cancer and Nutrition (EPIC). Cancer Epidemiol Biomarkers Prev (2011) 20:208–14. doi: 10.1158/1055-9965.EPI-10-0889 21098651

[23] Abe SK Inoue M Sawada N Iwasaki M Shimazu T Yamaji T. Hepatitis B and C Virus Infection and Risk of Lymphoid Malignancies: A Population-Based Cohort Study (JPHC Study). Cancer Epidemiol (2015) 39:562–6. doi: 10.1016/j.canep.2015.06.002 26149122

[24] Anaissie E Nucci M. Risks and Epidemiology of Infections After Autologous Stem Cell Transplantation. In: Bowden RA Ljungman P Paya CV, editors. Transplant Infections, 2nd ed. Philadelphia: Lippincott Williams & Wilkins (2003). p. 39–50.

[25] Miceli M Atoui R Thertulien R Barlogie B Anaissie E Walker R. Deep Septic Thrombophlebitis: An Unrecognized Cause of Relapsing Bacteremia in Patients With Cancer. J Clin Oncol (2004) 22:1529–31. doi: 10.1200/JCO.2004.99.289 15084634

[26] Knudsen LM Nielsen B Gimsing P Geisler C. Autologous Stem Cell Transplantation in Multiple Myeloma: Outcome in Patients With Renal Failure. Eur J Haematol (2005) 75:27–33. doi: 10.1111/j.1600-0609.2005.00446.x 15946307

[27] Miller MA. Clinical Management of Clostridium Difficile-Associated Disease. Clin Infect Dis (2007) 45(Suppl 2):S122–128. doi: 10.1086/519257 17683016

[28] Kataoka K Nannya Y Hangaishi A Imai Y Chiba S Takahashi T. Influence of Pretransplantation Serum Ferritin on Nonrelapse Mortality After Myeloablative and Nonmyeloablative Allogeneic Hematopoietic Stem Cell Transplantation. Biol Blood Marrow Transplant (2009) 15:195–204. doi: 10.1016/j.bbmt.2008.11.012 19167679

[29] Cordonnier C Maury S Pautas C Bastié JN Chehata S Castaigne S. Secondary Antifungal Prophylaxis With Voriconazole to Adhere to Scheduled Treatment in Leukemic Patients and Stem Cell Transplant Recipients. Bone Marrow Transplant (2004) 33:943–98. doi: 10.1038/sj.bmt.1704469 15034546

[30] Pelletier N Heyzer-Williams LJ Wong KA Urich E Fazilleau N Heyzer-Williams MG. Plasma Cells Negatively Regulate the Follicular Helper T Cell Program. Nat Immunol (2010) 11:1110–8. doi: 10.1038/ni.1954 PMC3058870 21037578

[31] Bermejo DA Jackson SW Gorosito-Serran M Acosta-Rodriguez EV Amezcua-Vesely MC Sather BD. Trypanosoma Cruzi Trans-Sialidase Initiates a Program Independent Pf the Transcripytion Factors RORgammat and AHR That Leads to IL-17 Production by Activated B Cells. Nat Immunol (2013) 14:514–22. doi: 10.1038/ni.2569 PMC3631452 23563688

[32] Matsumoto M Baba A Yokota T Nishikawa H Ohkawa Y Kayama H. Interleukin-10-Producing Plasmablasts Exert Regulatory Function in Autoimmune Inflammation. Immunity (2014) 41:1040–51. doi: 10.1016/j.immuni.2014.10.016 25484301

[33] Otsuki T Yata K Sakaguchi H Uno M Fujii T Wada H. IL-10 in Myeloma Cells. Leuk Lymphoma (2002) 43:969–74. doi: 10.1080/10428190290021579 12148907

[34] Kulkarni U Karsten CM Kohler T Hammerschmidt S Bommert K Tiburzy B. IL-10 Mediates Plasmacytosis-Associated Immunodeficiency by Inhibiting Complement-Mediated Neutrophil Migration. J Allergy Clin Immunol (2016) 137(5):1487–1497.e6. doi: 10.1016/j.jaci.2015.10.018 26653800

[35] Fessler BJ. Infectious Diseases in Systemic Lupus Erythematosus: Risk Factors, Management and Prophylaxis. Best Pract Res Clin Rheumatol (2002) 16:281–91. doi: 10.1053/berh.2001.0226 12041954

[36] Kuruvilla J Shepherd JD Sutherland HJ Nevill TJ Nitta J Le A. Long-Term Outcome of Myeloablative Allogeneic Stem Cell Transplantation for Multiple Myeloma. Biol Blood Marrow Transpl (2007) 13:925–31. doi: 10.1016/j.bbmt.2007.04.006 17640596

[37] Pratt G Goodyear O Moss P. Immunodeficiency and Immunotherapy in Multiple Myeloma. Br J Haematol (2007) 138:563–79. doi: 10.1111/j.1365-2141.2007.06705.x 17686051

[38] Merico F Bergui L Gregoretti MG Ghia P Aimo G Lindley IJD. Cytokines Involved in the Progression of Multiple Myeloma. Clin Exp Immunol (1993) 92:27–31. doi: 10.1111/j.1365-2249.1993.tb05943.x 8467562 PMC1554871

[39] Paiva B Azpilikueta A Puig N Ocio EM Sharma R Oyajobi BO. PD-L1/PD-1 Presence in the Tumor Microenvironment and Activity of PD-1 Blockade in Multiple Myeloma. Leukemia (2015) 29:2110–3. doi: 10.1038/leu.2015.79 25778100

[40] Braga WMT Vettore AL Carvalho AC Atanackovic D Colleoni GWB. Overexpression of CTLA-4 in the Bone Marrow of Patients With Multiple Myeloma As a Sign of Local Accumulation of Immunosuppressive Tregs –Perspectives for Novel Treatment Strategies. Blood (2011) 118:1829. doi: 10.1182/blood.V118.21.1829.1829

[41] Tamura H Ishibashi M Yamashita T Tanosaki S Okuyama N Kondo A. Marrow Stromal Cells Induce B7-H1 Expression on Myeloma Cells, Generating Aggressive Characteristics in Multiple Myeloma. Leukemia (2013) 27:464–72. doi: 10.1038/leu.2012.213 22828443

[42] Ray A Das DS Song Y Richardson P Munshi NC Chauhan D. Targeting PD1-PDL1 Immune Checkpoint in Plasmacytoid Dendritic Cells Interactions With T Cells, Natural Killer Cells, and Multiple Myeloma Cells. Leukemia (2015) 29:1441–4. doi: 10.1038/leu.2015.11 PMC5703039 25634684

[43] Romano A Conticello C Cavalli M Vetro C La Fauci A Parrinello NL. Immunological Dysregulation in Multiple Myeloma Microenvironment. BioMed Res Int (2014) 2014:198539. doi: 10.1155/2014/198539 25013764 PMC4071780

[44] Allegra A Di Gioacchino M Tonacci A Musolino C Gangemi S. Immunopathology of SARS-CoV-2 Infection: Immune Cells and Mediators, Prognostic Factors, and Immune-Therapeutic Implications. Int J Mol Sci (2020) 21(13):4782. doi: 10.3390/ijms21134782 PMC7370171 32640747

[45] Fourcade J Sun Z Benallaoua M Guillaume P Luescher IF Sander C. Upregulation of Tim-3 and PD-1 Expression is Associated With Tumor Antigen-Specific CD8+ T Cell Dysfunction in Melanoma Patients. J Exp Med (2010) 207:2175–86. doi: 10.1084/jem.20100637 PMC2947081 20819923

[46] Anderson AC Joller N Kuchroo VK. Lag-3, Tim-3, and TIGIT: Coinhibitory Receptors With Specialized Functions in Immune Regulation. Immunity (2016) 44:989–1004. doi: 10.1016/j.immuni.2016.05.001 27192565 PMC4942846

[47] Zhou Q Munger ME Veenstra RG Weigel BJ Hirashima M Munn DH. Coexpression of Tim-3 and PD-1 Identifies a CD8(+) T-Cell Exhaustion Phenotype in Mice With Disseminated Acute Myelogenous Leukemia. Blood (2011) 117:4501–10. doi: 10.1182/blood-2010-10-310425 PMC3099570 21385853

[48] Tan J Chen S Lu Y Yao D Xu L Zhang Y. Higher PD-1 Expression Concurrent With Exhausted CD8+ T Cells in Patients With *De Novo* Acute Myeloid Leukemia. Chin J Cancer Res (2017) 29:463–70. doi: 10.1016/j.exphem.2017.06.190 PMC5677131 29142466

[49] Chung DJ Pronschinske KB Shyer JA Sharma S Leung S Curran SA. T-Cell Exhaustion in Multiple Myeloma Relapse After Autotransplant: Optimal Timing of Immunotherapy. Cancer Immunol Res (2016) 4:61–71. doi: 10.1158/2326-6066.CIR-15-0055 26464015 PMC4703436

[50] Tan J Chen S Huang J Chen Y Yang L Wang C. Increased Exhausted CD8^+^ T Cells With Programmed Death-1, T-Cell Immunoglobulin and Mucin-Domain-Containing-3 Phenotype in Patients With Multiple Myeloma. Asia Pac J Clin Oncol (2018) 14(5):e266–74. doi: 10.1111/ajco.13033 29943497

[51] Kumar H Kawai T Akira S. Pathogen Recognition by the Innate Immune System. Int Rev Immunol (2011) 30:16–34. doi: 10.3109/08830185.2010.529976 21235323

[52] Takeda K Akira S. Toll Receptors and Pathogen Resistance. Cell Microbiol (2003) 5:143–53. doi: 10.1046/j.1462-5822.2003.00264.x 12614458

[53] Beutler BA. TLRs and Innate Immunity. Blood (2009) 113:1399–407. doi: 10.1182/blood-2008-07-019307 PMC2644070 18757776

[54] Akira S. Mammalian Toll-Like Receptors. Curr Opin Immunol (2003) 15:5–11. doi: 10.1016/S0952-7915(02)00013-4 12495726

[55] Facon T Mary JY Pegourie B Attal M Renaud M Sadoun A. Dexamethasone-Based Regimens *Versus* Melphalan-Prednisone for Elderly Multiple Myeloma Patients Ineligible for High-Dose Therapy. Blood (2005) 107:1292–8. doi: 10.1182/blood-2005-04-1588 16174762

[56] Shustik C Belch A Robinson S Rubin SH Dolan SP Kovacs MJ. Randomised Comparison of Melphalan With Prednisone or Dexamethasone as Induction Therapy and Dexamethasone or Observation as Maintenance Therapy in Multiple Myeloma: NCIC CTG MY.7. Br J Haematol (2007) 136:203–11. doi: 10.1111/j.1365-2141.2006.06405.x 17233817

[57] Rajkumar SV Jacobus S Callander N Fonseca R Vesole D Williamns M. Randomized Trial of Lenalidomide Plus High-Dose Dexamethasone *Versus* Lenalidomide Plus Low-Dose Dexamethasone in Newly Diagnosed Myeloma (E4A03), a Trial Coordinated by the Eastern Cooperative Oncology Group: Analysis of Response. Blood (2007) 110:30a. doi: 10.1182/blood.V110.11.74.74

[58] Sun K Welniak LA Panoskaltsis-Mortari A O’Shaughnessy MJ Liu H Barao I. Inhibition of Acute Graft-*Versus*-Host Disease With Retention of Graft-*Versus*-Tumor Effects by the Proteasome Inhibitor Bortezomib. Proc Natl Acad Sci U S A (2004) 101:8120–5. doi: 10.1073/pnas.0401563101 PMC419567 15148407

[59] Uy GL Peles S Fisher NM Tomasson MH DiPersio JF Vij R. Bortezomib Prior to Autologous Transplant in Multiple Myeloma: Effects in Mobilization, Engraftment, and Markers of Immune Function. Biol Blood Marrow Transplant (2006) 12(Suppl 1):116. doi: 10.1016/j.bbmt.2005.11.355

[60] Richardson P Mitsiades C Schlossman R Ghobrial I Hideshima T Chauhan D. The Treatment of Relapsed and Refractory Multiple Myeloma. Hematol Am Soc Hematol Educ Program (2007) 2007:317–23. doi: 10.1182/asheducation-2007.1.317 18024646

[61] Facon T Mary JY Hulin C Benboubker L Attal M Pegourie B. Melphalan and Prednisone Plus Thalidomide *Versus* Melphalan and Prednisone Alone or Reduced-Intensity Autologous Stem Cell Transplantation in Elderly Patients With Multiple Myeloma (IFM 99–06): A Randomised Trial. Lancet (2007) 370:1209–18. doi: 10.1016/S0140-6736(07)61537-2 17920916

[62] Marriott JB Dredge K Dalgleish AG. Thalidomide Derived Immunomodulatory Drugs (IMiDs) as Potential Therapeutic Agents. Curr Drug Targets Immune Endocr Metabol Disord (2003) 3):181–6. doi: 10.2174/1568008033340207 12871024

[63] Rajkumar SV Hayman SR Lacy MQ Dispenzieri A Geyer SM Kabat B. Combination Therapy With Lenalidomide Plus Dexamethasone (REV/DEX) for Newly Diagnosed Myeloma. Blood (2005) 106:4050–3. doi: 10.1182/blood-2005-07-2817 PMC1895238 16118317

[64] San Miguel JF Schlag R Khuageva NK Dimopoulos MA Shpilberg O Kropff M. Bortezomib Plus Melphalan and Prednisone for Initial Treatment of Multiple Myeloma. N Engl J Med (2008) 359:906–17. doi: 10.1056/NEJMoa0801479 18753647

[65] D’Avignon LC Schofield CM Hospenthal DR. *Pneumocystis* Pneumonia. Semin Respir Crit Care Med (2008) 29:132–40. doi: 10.1055/s-2008-1063852 18365995

[66] Blanco B Perez-Simon JA Sanchez-Abarca LI Carvajal-Vergara X Mateos J Vidriales B. Bortezomib Induces Selective Depletion of Alloreactive T Lymphocytes and Decreases the Production of Th1 Cytokines. Blood (2006) 107:3575–83. doi: 10.1182/blood-2005-05-2118 16282346

[67] Miceli MH Dong L Grazziutti ML Fassas A Thertulien R Van Rhee F. Iron Overload is a Major Risk Factor for Severe Infection After Autologous Stem Cell Transplantation: A Study of 367 Myeloma Patients. Bone Marrow Transplant (2006) 37:857–64. doi: 10.1038/sj.bmt.1705340 16532017

[68] Tsukayama DT. Pathophysiology of Posttraumatic Osteomyelitis. Clin Orthop Relat Res (1999) 360:22–9. doi: 10.1097/00003086-199903000-00005 10101307

[69] Dispenzieri A. Complications of Myeloma Therapy. Hematol Oncol Clin North Am (2007) 21:1247–73. doi: 10.1016/j.hoc.2007.08.002 17996597

[70] Rayner HC Haynes AP Thompson JR Russell N Fletcher J. Perspectives in Multiple Myeloma: Survival, Prognostic Factors and Disease Complications in a Single Centre Between 1975 and 1988. Q J Med (1991) 79:517–25. 1946932

[71] Wang M Dimopoulos MA Chen C Cibeira MT Attal M Spencer A. Lenalidomide Plus Dexamethasone is More Effective Than Dexamethasone Alone in Patients With Relapsed or Refractory Multiple Myeloma Regardless of Prior Thalidomide Exposure. Blood (2008) 112:4445–51. doi: 10.1182/blood-2008-02-141614 18799726

[72] Coxon FP Thompson K Rogers MJ. Recent Advances in Understanding the Mechanism of Action of Bisphosphonates. Curr Opin Pharmacol (2006) 6:307–12. doi: 10.1016/j.coph.2006.03.005 16650801

[73] Chadwick JW Tenenbaum HC Sun CX Wood RE Glogauer M. The Effect of Pamidronate Delivery in Bisphosphonate-Naïve Patients on Neutrophil Chemotaxis and Oxidative Burst. Sci Rep (2020) 10(1):18309. doi: 10.1038/s41598-020-75272-6 33110111 PMC7591900

[74] Badros A Weikel D Salama A Goloubeva O Schneider A Rapoport A. Osteonecrosis of the Jaw in Multiple Myeloma Patients: Clinical Features and Risk Factors. J Clin Oncol (2006) 24:945–52. doi: 10.1200/JCO.2005.04.2465 16484704

[75] Alonci A Allegra A Bellomo G Quartarone E Oteri G Nastro E. Patients With Bisphosphonate-Associated Osteonecrosis of the Jaw Have Unmodified Levels of Soluble Vascular Endothelial Growth Factor Receptor 1. Leuk Lymphoma (2007) 48(9):1852–4. doi: 10.1080/10428190701509806 17786724

[76] Rajkumar SV Blood E Vesole D Fonseca R Greipp PR. Phase III Clinical Trial of Thalidomide Plus Dexamethasone Compared With Dexamethasone Alone in Newly Diagnosed Multiple Myeloma: A Clinical Trial Coordinated by the Eastern Cooperative Oncology Group. J Clin Oncol (2006) 24:431–6. doi: 10.1200/JCO.2005.03.0221 16365178

[77] Clarke BM Boyette J Vural E Suen JY Anaissie EJ Stack BC Jr. Bisphosphonates and Jaw Osteonecrosis: The UAMS Experience. Otolaryngol Head Neck Surg (2007) 136:396–400. doi: 10.1016/j.otohns.2006.11.008 17321866

[78] Khan AA Sandor GK Dore E Morrison AD Alsahli M Amin F. Canadian Consensus Practice Guidelines for Bisphosphonate Associated Osteonecrosis of the Jaw. J Rheumatol (2008) 35:1391–7. 18528958

[79] Chapel HM Lee M Hargreaves R Pamphilon DH Prentice AG. Randomised Trial of Intravenous Immunoglobulin as Prophylaxis Against Infection in Plateau-Phase Multiple Myeloma. The UK Group for Immunoglobulin Replacement Therapy in Multiple Myeloma. Lancet (1994) 343(8905):1059–63. doi: 10.1016/S0140-6736(94)90180-5 7909099

[80] Ye C Chen W Gao Q Chen Y Song X Zheng S. Secondary Immunodeficiency and Hypogammaglobulinemia With IgG Levels of <5 G/L in Patients With Multiple Myeloma: A Retrospective Study Between 2012 and 2020 at a University Hospital in China. Med Sci Monit (2021) 27:e930241. doi: 10.12659/MSM.930241 34238914 PMC8276618

[81] Karlsson J Andreasson B Kondori N Erman E Riesbeck K Hogevik H. Comparative Study of Immune Status to Infectious Agents in Elderly Patients With Multiple Myeloma, Waldenstrom’s Macroglobulinemia, and Monoclonal Gammopathy of Undetermined Significance. Clin Vaccine Immunol (2011) 18(6):969–77. doi: 10.1128/CVI.00021-11 PMC3122605 21508164

[82] Vesole DH Oken MM Heckler C Greipp PR Katz MS Jacobus S. Oral Antibiotic Prophylaxis of Early Infection in Multiple Myeloma: A URCC/ECOG Randomized Phase III Study. Leukemia (2012) 26:2517–20. doi: 10.1038/leu.2012.124 PMC4734137 22678167

[83] Lavi N Avivi I Kra-Oz Z Oren I Hardak E. Community-Acquired Respiratory Infections are Common in Patients With non-Hodgkin Lymphoma and Multiple Myeloma. Support Care Cancer (2018) 26:2425–31. doi: 10.1007/s00520-018-4079-3 29427192

[84] Vickrey E Allen S Mehta J. Singhal Acyclovir to Prevent Reactivation of Varicella Zoster Virus (Herpes Zoster) in Multiple Myeloma Patients Receiving Bortezomib Therapy. Cancer (2015) 115:229–32. doi: 10.1002/cncr.24006 19090004

[85] Satlin MJ Vardhana S Soave R Shore TB Mark TM Jacobs SE. Impact of Prophylactic Levofloxacin on Rates of Bloodstream Infection and Fever in Neutropenic Patients With Multiple Myeloma Undergoing Autologous Hematopoietic Stem Cell Transplantation. Biol Blood Marrow Transpl (2015) 21:1808–14. doi: 10.1016/j.bbmt.2015.06.017 PMC4568152 26150022

[86] Eriksson T Bjorkman S Hoglund P. Clinical Pharmacology of Thalidomide. Eur J Clin Pharmacol (2001) 57:365–76. doi: 10.1007/s002280100320 11599654

[87] Leveque D Carvalho MC Maloisel F. Review. Clinical Pharmacokinetics of Bortezomib. *In Vivo* (2007) 21:273–8. 17436576

[88] Drayson MT Bowcock S Planche T Iqbal G Pratt G Yong K. TEAMM Trial Management Group and Trial Investigators. Levofloxacin Prophylaxis in Patients With Newly Diagnosed Myeloma (TEAMM): A Multicentre, Double-Blind, Placebo-Controlled, Randomised, Phase 3 Trial. Lancet Oncol (2019) 20(12):1760–72. doi: 10.1016/S1470-2045(19)30506-6 PMC6891230 31668592

[89] Valkovic T Nacinovic AD Petranovic D. Prophylactic Broad Spectrum Antibiotics as a New Anti-Myeloma Therapy. Med Hypotheses (2013) 81:1137–40. doi: 10.1016/j.mehy.2013.10.021 24238832

[90] Wadhwa PD Morrison VA. Infectious Complications of Chronic Lymphocytic Leukemia. Semin Oncol (2006) 33:240–9. doi: 10.1053/j.seminoncol.2005.12.013 16616071

[91] Dhalla F Lucas M Schuh A Bhole M Jain R Patel SY. Antibody Deficiency Secondary to Chronic Lymphocytic Leukemia: Should Patients be Treated With Prophylactic Replacement Immunoglobulin? J Clin Immunol (2014) 34:277–82. doi: 10.1007/s10875-014-9995-5 24557494

[92] Hallek M. Chronic Lymphocytic Leukemia: 2015 Update on Diagnosis, Risk Stratification, and Treatment. Am J Hematol (2015) 90:446–60. doi: 10.1002/ajh.23979 25908509

[93] Friman V Winqvist O Blimark C Langerbeins P Chapel H Dhalla F. Secondary Immunodeficiency in Lymphoproliferative Malignancies. Hematol Oncol (2016) 34:121–32. doi: 10.1002/hon.2323 27402426

[94] Shimoni A Marcus H Canaan A Ergas D David M Berrebi A. A Model for Human B-Chronic Lymphocytic Leukemia in Human/Mouse Radiation Chimera: Evidence for Tumor-Mediated Suppression of Antibody Production in Low-Stage Disease. Blood (1997) 89(6):2210–8. doi: 10.1182/blood.V89.6.2210 9058746

[95] Forconi F Moss P. Perturbation of the Normal Immune System in Patients With CLL. Blood (2015) 126(5):573–81. doi: 10.1182/blood-2015-03-567388 26084672

[96] Bussel JB Cunningham-Rundles C. Intravenous Usage of Gammaglobulin: Humoral Immunodeficiency, Immune Thrombocytopenic Purpura, and Newer Indications. Cancer Invest (1985) 3(4):361–6. doi: 10.31099/07357908509039797 4040795

[97] Cantwell M Hua T Pappas J Kipps TJ. Acquired CD40-Ligand Deficiency in Chronic Lymphocytic Leukemia. Nat Med (1997) 3(9):984–9. doi: 10.1038/nm0997-984 9288724

[98] Cerutti A Kim EC Shah S Schattner EJ Zan H Schaffer A. Dysregulation of CD30+ T Cells by Leukemia Impairs Isotype Switching in Normal B Cells. Nat Immunol (2001) 2(2):150–6. doi: 10.1038/84254 PMC4621971 11175813

[99] Hamblin AD. Hamblin TJ The Immunodeficiency of Chronic Lymphocytic Leukaemia. Br Med Bull (2008) 87:49–62. doi: 10.1093/bmb/ldn034 18755702

[100] Grywalska E Zaborek M Łyczba J Hrynkiewicz R Bębnowska D Becht R. Chronic Lymphocytic Leukemia-Induced Humoral Immunosuppression: A Systematic Review. Cells (2020) 9(11):2398. doi: 10.3390/cells9112398 PMC7693361 33147729

[101] Phillips AC Carroll D Burns VE Ring C Macleod J Drayson M. Bereavement and Marriage are Associated With Antibody Response to Influenza Vaccination in the Elderly. Brain Behav Immun (2006) 20:279–89. doi: 10.1016/j.bbi.2005.08.003 16198083

[102] Parry HM Birtwistle J Whitelegg A Hudson C McSkeane T Hazelwood P. Poor Functional Antibody Responses are Present in Nearly All Patients With Chronic Lymphocytic Leukaemia, Irrespective of Total IgG Concentration, and are Associated With Increased Risk of Infection. Br J Haematol (2015) 171(5):887–90. doi: 10.1111/bjh.13455 25920967

[103] Oscier D Dearden C Eren E Fegan C Follows G Hillmen P. Use and Interpretation of Diagnostic Vaccination in Primary Immunodeficiency: A Working Group Report of the Basic and Clinical Immunology Interest Section of the American Academy of Allergy, Asthma & Immunology. J Allergy Clin Immunol (2012) 130(3 Suppl):S1–S24. doi: 10.1016/j.jaci.2012.07.002 22935624

[104] Paris K Sorensen RU. Assessment and Clinical Interpretation of Polysaccharide Antibody Responses. Ann Allergy Asthma Immunol (2007) 99:462–4. doi: 10.1016/S1081-1206(10)60572-8 18051217

[105] Fust G Czink E Minh D Miszlay Z Varga L Hollan SR. Depressed Classical Complement Pathway Activities in Chronic Lymphocytic Leukaemia. Clin Exp Immunol (1985) 60(3):489–95. PMC1577204 4017286

[106] Arruga F Gyau BB Iannello A Vitale N Vaisitti T Deaglio S. Immune Response Dysfunction in Chronic Lymphocytic Leukemia: Dissecting Molecular Mechanisms and Microenvironmental Conditions. Int J Mol Sci (2020) 21:1825. doi: 10.3390/ijms21051825 PMC7084946 32155826

[107] Riches JC Ramsay AG Gribben JG. Immune Reconstitution in Chronic Lymphocytic Leukemia. Curr Hematol Malig Rep (2012) 7:13–20. doi: 10.1007/s11899-011-0106-x 22231031 PMC4533917

[108] Nosari A. Infectious Complications in Chronic Lymphocytic Leukemia. Mediterr J Hematol Infect Dis (2012) 4:e2012070. doi: 10.4084/mjhid.2012. 23205258 PMC3507529

[109] Bhattacharya N Diener S Idler IS Rauen J Häbe S Busch H. Nurse-Like Cells Show Deregulated Expression of Genes Involved in Immunocompetence. Br J Haematol (2011) 154(3):349–56. doi: 10.1111/j.1365-2141.2011.08747.x 21615384

[110] Riches JC Gribben JG. Understanding the Immunodeficiency in Chronic Lymphocytic Leukemia: Potential Clinical Implications. Hematol Oncol Clin North Am (2013) 27(2):207–35. doi: 10.1016/j.hoc.2013.01.003 23561470

[111] Christopoulos P Pfeifer D Bartholomé K Follo M Timmer J Fisch P. Definition and Characterization of the Systemic T-Cell Dysregulation in Untreated Indolent B-Cell Lymphoma and Very Early CLL. Blood (2011) 117(14):3836–46. doi: 10.1182/blood-2010-07-299321 21270444

[112] Gorgun G Holderried TA Zahrieh D Neuberg D Gribben JG. Chronic Lymphocytic Leukemia Cells Induce Changes in Gene Expression of CD4 and CD8 T Cells. J Clin Invest (2005) 115(7):1797–805. doi: 10.1172/JCI24176 PMC1150284 15965501

[113] Ramsay AG Clear AJ Kelly G Fatah R Matthews J Macdougall F. Follicular Lymphoma Cells Induce T-Cell Immunologic Synapse Dysfunction That can be Repaired With Lenalidomide: Implications for the Tumor Microenvironment and Immunotherapy. Blood (2009) 114(21):4713–20. doi: 10.1182/blood-2009-04-217687 PMC2780306 19786615

[114] Ramsay AG Johnson AJ Lee AM Gorgün G Le Dieu R Blum W. Chronic Lymphocytic Leukemia T Cells Show Impaired Immunological Synapse Formation That can be Reversed With an Immunomodulating Drug. J Clin Invest (2008) 118(7):2427–37. doi: 10.1172/JCI35017 PMC2423865 18551193

[115] Beyer M Kochanek M Darabi K Popov A Jensen M Endl E. Reduced Frequencies and Suppressive Function of CD4_CD25hi Regulatory T Cells in Patients With Chronic Lymphocytic Leukemia After Therapy With Fludarabine. Blood (2005) 106(6):2018–25. doi: 10.1182/blood-2005-02-0642 15914560

[116] Montironi C Muñoz-Pinedo C Eldering E. Hematopoietic *Versus* Solid Cancers and T Cell Dysfunction: Looking for Similarities and Distinctions. Cancers (Basel) (2021) 13(2):284. doi: 10.3390/cancers13020284 33466674 PMC7828769

[117] Aittoniemi J Miettinen A Laine S Sinisalo M Laippala P Vilpo L. Opsonising Immunoglobulins and Mannan-Binding Lectin in Chronic Lymphocytic Leukemia. Leuk Lymphoma (1999) 34(3–4):381–5. doi: 10.3109/10428199909050963 10439375

[118] Itala M Vainio O Remes K. Functional Abnormalities in Granulocytes Predict Susceptibility to Bacterial Infections in Chronic Lymphocytic Leukaemia. *Eur J Haematol* (1996) 57(1):46–53. doi: 10.1111/j.1600-0609.1996.tb00489.x 8698131

[119] Jurado-Camino T Cordoba R Esteban-Burgos L Hernandez-Jimenez E Toledano V Hernandez-Rivas JA. Chronic Lymphocytic Leukemia: A Paradigm of Innate Immune Cross-Tolerance. J Immunol (2015) 194(2):719–27. doi: 10.4049/jimmunol.1402272 25505275

[120] Hensel M Kornacker M Yammeni S Egerer G Ho AD. Disease Activity and Pretreatment, Rather Than Hypogammaglobulinaemia, are Major Risk Factors for Infectious Complications in Patients With Chronic Lymphocytic Leukaemia. Br J Haematol (2003) 122(4):600–6. doi: 10.1046/j.1365-2141.2003.04497.x 12899715

[121] Ravandi F O’Brien S. Immune Defects in Patients With Chronic Lymphocytic Leukemia. Cancer Immunol Immunother (2006) 55(2):197–209. doi: 10.1007/s00262-005-0015-8 16025268 PMC11029864

[122] Anderson D Ali K Blanchette V Brouwers M Couban S Radmoor P. Guidelines on the Use of Intravenous Immune Globulin for Hematologic Conditions. Transfus Med Rev (2007) 21(2 Suppl 1):S9–56. doi: 10.1016/j.tmrv.2007.01.001 17397769

[123] Brinkmann V Reichard U Goosmann C Fauler B Uhlemann Y Weiss DS. Neutrophil Extra-Cellular Traps Kill Bacteria. Science (2004) 303:1532–5. doi: 10.1126/science.1092385 15001782

[124] Tinhofer I Marschitz I Kos M Henn T Egle A Villunger A. Differential Sensitivity of CD4+ and CD8+ T Lymphocytes to the Killing Efficacy of Fas (Apo-1/CD95) Ligand+ Tumor Cells in B Chronic Lymphocytic Leukemia. Blood (1988) 91:4273–81. doi: 10.1182/blood.V91.11.4273.411k25_4273_4281 9596676

[125] Sampalo A Navas G Medina F Segundo C Cámara C Brieva JA. Chronic Lymphocytic Leukemia B Cells Inhibit Spontaneous Ig Production by Autologous Bone Marrow Cells: Role of CD95-CD95L Interaction. Blood (2005) 96:3168–74. doi: 10.1182/blood.V96.9.3168.h8003168_3168_3174 11049999

[126] Patel SY Carbone J Jolles S. The Expanding Field of Secondary Antibody Deficiency: Causes, Diagnosis, and Management. Front Immunol (2019) 10:33. doi: 10.3389/fimmu.2019.00033 30800120 PMC6376447

[127] Yap PL. Applications of Intravenous Immunoglobulin. Malays J Pathol (1990) 12(1):1–10. 2090883

[128] Compagno N Malipiero G Cinetto F Agostini C. Immunoglobulin Replacement Therapy in Secondary Hypogammaglobulinemia. Front Immunol (2014) 5:626. doi: 10.3389/fimmu.2014.00626 25538710 PMC4259107

[129] Compagno N Cinetto F Semenzato G Agostini C. Subcutaneous Immunoglobulin in Lymphoproliferative Disorders and Rituximab-Related Secondary Hypogammaglobulinemia: A Single-Center Experience in 61 Patients. Haematologica (2014) 99:1101–6. doi: 10.3324/haematol.2013.101261 PMC4040915 24682509

[130] Jolles S Michallet M Agostini C Albert MH Edgar D Ria R. Treating Secondary Antibody Deficiency in Patients With Haematological Malignancy: European Expert Consensus. Eur J Haematol (2021) 106(4):439–49. doi: 10.1111/ejh.13580 33453130

[131] Jolles S Orange JS Gardulf A Stein MR Shapiro R Borte M. Current Treatment Options With Immunoglobulin G for the Individualization of Care in Patients With Primary Immunodeficiency Disease. Clin Exp Immunol (2015) 179:146–60. doi: 10.1111/cei.12485 PMC4298393 25384609

[132] Sewell WA Kerr J Behr-Gross ME Peter HH Kreuth Ig Working Group. European Consensus Proposal for Immunoglobulin Therapies. Eur J Immunol (2014) 44:2207–14. doi: 10.1002/eji.201444700 24975475

[133] Octapharma Canada Inc. PANZYGA® Product Monograph (2017). Available at: https://www.octapharma.ca/fileadmin/user_upload/octapharma.ca/Product_Monographs/PANZYGA-PM-EN.pdf (Accessed September 07, 2018).

[134] 178. Grifols Canada Ltd. FLEBOGAMMA® 10% Product Monograph Barcelona, Spain.

[135] European Medicines Agency. Guideline on Core SmPC for Human Normal Immunoglobulin for Subcutaneous and Intramuscular Administration (2015). Available at: http://www.ema.europa.eu/docs/en_GB/document_library/Scientific_guideline/2015/03/WC500184870.pdf (Accessed May 14, 2018).

[136] European Medicines Agency. Guideline on Core SmPC for Human Normal Immunoglobulin for Intravenous Administration (IVIg) (2018). Available at: http://www.ema.europa.eu/docs/en_GB/document_library/.

[137] Baxalta US Inc. GAMMAGARD s/D Full Prescribing Information (2017). Available at: https://www.fda.gov/downloads/BloodBloodProducts/UCM197905.pdf (Accessed May 17, 2018).

[138] Na IK Buckland M Agostini C Edgar JDM Friman V Michallet M. Current Clinical Practice and Challenges in the Management of Secondary Immunodeficiency in Hematological Malignancies. Eur J Haematol (2019) 102(6):447–56. doi: 10.1111/ejh.13223 PMC6849602 30801785

[139] Parikh SA Leis JF Chaffee KG Call TG Hanson CA Ding W. Hypogammaglobulinemia in Newly Diagnosed Chronic Lymphocytic Leukemia: Natural History, Clinical Correlates, and Outcomes. Cancer (2015) 121(17):2883–91. doi: 10.1002/cncr.29518 PMC4545721 25931291

[140] Weeks JC Tierney MR Weinstein MC. Cost Effectiveness of Prophylactic Intravenous Immune Globulin in Chronic Lymphocytic Leukemia. N Engl J Med (1991) 325(2):81–6. doi: 10.1056/NEJM199107113250202 1904989

[141] European Medicines Agency. Guideline on the Clinical Investigation of Human Normal Immunoglobulin for Intravenous Administration (IVIg). EMA (2018). Available at: https://www.ema.europa.eu/documents/scientific-guideline/guideline-clinical-investigation-human-normalimmunoglobulin-intravenous-administration-ivig-rev-3_en.pdf.

[142] Streu E Banerji V Dhaliwal DHS. The Efficacy and Cost Effectiveness of Subcutaneous Immunoglobulin (SCIG) Replacement in Patients With Immune Deficiency Secondary to Chronic Lymphocytic Leukemia. Blood (2016) 128:4778. doi: 10.1182/blood.V128.22.4778.4778

[143] Eichhorst B Robak T Montserrat E Ghia P Hillmen P Hallek M. Et al. Chronic Lymphocytic Leukaemia: ESMO Clinical Practice Guidelines for Diagnosis, Treatment and Follow-Up. Ann Oncol (2015) 26(Suppl 5):78–84. doi: 10.1093/annonc/mdv233.265 33091559

[144] Obeid KM Aguilar J Szpunar S Sharma M del Busto R Al-Katib A. Risk Factors for Pneumocystis Jirovecii Pneumonia in Patients With Lymphoproliferative Disorders. Clin Lymphoma Myeloma Leuk (2012) 12(1):66–9. doi: 10.1016/j.clml.2011.07.006 22000698

[145] Allegra A Innao V Gerace D Vaddinelli D Musolino C. Adoptive Immunotherapy for Hematological Malignancies: Current Status and New Insights in Chimeric Antigen Receptor T Cells. Blood Cells Mol Dis (2016) 62:49–63. doi: 10.1016/j.bcmd.2016.11.001 27865176

[146] Casulo C Maragulia J Zelenetz AD. Incidence of Hypogammaglobulinemia in Patients Receiving Rituximab and the Use of Intravenous Immunoglobulin for Recurrent Infections. Clin Lymphoma Myeloma Leuk (2013) 13:106–11. doi: 10.1016/j.clml.2012.11.011 PMC4035033 23276889

[147] Besada E Bader L Nossent H. Sustained Hypogammaglobulinemia Under Rituximab Maintenance Therapy Could Increase the Risk for Serious Infections: A Report of Two Cases. Rheumatol Int (2013) 33(6):1643–4. doi: 10.1007/s00296-012-2483-4 22207199

[148] Visco C Finotto S Pomponi F Sartori R Laveder F Trentin L. The Combination of Rituximab, Bendamustine, and Cytarabine for Heavily Pre-Treated Relapsed/Refractory Cytogenetically High-Risk Patients With Chronic Lymphocytic Leukemia. Am J Hematol (2013) 88(4):289–93. doi: 10.1002/ajh.23396 23450436

[149] De Angelis F Tosti ME Capria S Russo E D’Elia GM Annechini G. Risk of Secondary Hypogammaglobulinaemia After Rituximab and Fludarabine in Indolent non-Hodgkin Lymphomas: A Retrospective Cohort Study. Leuk Res (2015) 39(12):1382–8. doi: 10.1016/j.leukres.2015.10.013 26547259

[150] Tuccori M Focosi D Blandizzi C Pelosini M Montagnani S Maggi F. Inclusion of Rituximab in Treatment Protocols for non-Hodgkin’s Lymphomas and Risk for Progressive Multifocal Leukoencephalopathy. Oncologist (2010) 15(11):1214–9. doi: 10.1634/theoncologist.2010-0098 PMC3227905 21041380

[151] Ozoya OO Sokol L Dalia S. Hepatitis B Reactivation With Novel Agents in non-Hodgkin’s Lymphoma and Prevention Strategies. J Clin Transl Hepatol (2016) 4(2):143–50. doi: 10.14218/JCTH.2016.00005 PMC4913070 27350944

[152] Morrison VA. Infectious Complications in Patients With Chronic Lymphocytic Leukemia: Pathogenesis, Spectrum of Infection, and Approaches to Prophylaxis. Clin Lymphoma Myeloma (2009) 9(5):365–70. doi: 10.3816/CLM.2009.n.071 19858055

[153] Kimby E. Tolerability and Safety of Rituximab (MabThera). Cancer Treat Rev (2005) 31:456–73. doi: 10.1016/j.ctrv.2005.05.007 16054760

[154] Castagnola E Dallorso S Faraci M Morreale G Di Martino D Cristina E. Long-Lasting Hypogammaglobulinemia Following Rituximab Administration for Epstein-Barr Virus-Related Post-Transplant Lymphoproliferative Disease Preemptive Therapy. J Hematother Stem Cell Res (2003) 12(1):9–10. doi: 10.1089/152581603321210082 12662431

[155] Cooper N Davies EG. Thrasher AJ Repeated Courses of Rituximab for Autoimmune Cytopenias may Precipitate Profound Hypogammaglobulinaemia Requiring Replacement Intravenous Immunoglobulin. Br J Haematol (2009) 46:120–2. doi: 10.1111/j.1365-2141.2009.07715.x 19438506

[156] Wolach O Bairey O Lahav M. Late-Onset Neutropenia After Rituximab Treatment: Case Series and Comprehensive Review of 12, the Literature. Medicine (2010) 89:308–18. doi: 10.1097/MD.0b013e3181f2caef 20827108

[157] Aksoy S Harputluoglu H Kilickap S Dede DS Dizdar O Altundag K. Rituximab-Related Viral Infections in Lymphoma Patients. Leuk Lymphoma (2007) 48:1307–12. doi: 10.1080/10428190701411441 17613758

[158] Le Clech L Ianotto J-C Quintin-Roue I Tempescul A. Severe CMV Complication Following Maintenance Therapy With Rituximab. BMJ Case Rep (2013) 2013:bcr2012006672. doi: 10.1136/bcr-2012-006672 PMC3603828 23307449

[159] Carson KR Evens AM Richey EA Habermann TM Focosi D Seymour JF. Progressive Multifocal Leukoencephalopathy After Rituximab Therapy in HIV-Negative Patients: A Report of 57 Cases From the Research on Adverse Drug Events and Reports Project. Blood (2009) 113:4834–40. doi: 10.1182/blood-2008-10-186999 PMC2686134 19264918

[160] van der Kolk LE Baars JW Prins MH Oers v. MH Rituximab Treatment Results in Impaired Secondary Humoral Immune Responsiveness. Blood (2002) 100:2257–9. doi: 10.1182/blood.V100.6.2257.h81802002257_2257_2259 12200395

[161] Diwakar L Gorrie S Richter A Chapman O Dhillon P Al-Ghanmi F. Does Rituximab Aggravate Pre-Existing Hypogammaglobulinaemia? J Clin Pathol (2010) 63:275–7. doi: 10.1136/jcp.2009.068940 20203231

[162] Makatsori M Kiani-Alikhan S Manson AL Verma N Leandro M Gurugama NP. Hypogammaglobulinaemia After Rituximab Treatment-Incidence and Outcomes. QJM (2014) 107(10):821–8. doi: 10.1093/qjmed/hcu094 24778295

[163] Byrd JC Harrington B O’Brien S Jones JA Schuh A Devereux S. Acalabrutinib (ACP-196) in Relapsed Chronic Lymphocytic Leukemia. N Engl J Med (2016) 374(4):323–32. doi: 10.1056/NEJMoa1509981 PMC4862586 26641137

[164] Burger JA Tedeschi A Barr PM Robak T Owen C Ghia P. Ibrutinib as Initial Therapy for Patients With Chronic Lymphocytic Leukemia. N Engl J Med (2015) 373(25):2425–37. doi: 10.1056/NEJMoa1509388 PMC4722809 26639149

[165] Furman RR Sharman JP Coutre SE Cheson BD Pagel JM Hillmen P. Idelalisib and Rituximab in Relapsed Chronic Lymphocytic Leukemia. N Engl J Med (2014) 370(11):997–1007. doi: 10.1056/NEJMoa1315226 24450857 PMC4161365

[166] Gopal AK Kahl BS de Vos S Wagner-Johnston ND Schuster SJ Jurczak WJ. PI3Kdelta Inhibition by Idelalisib in Patients With Relapsed Indolent Lymphoma. N Engl J Med (2014) 370(11):1008–18. doi: 10.1056/NEJMoa1314583 PMC4039496 24450858

[167] O’Brien SM Lamanna N Kipps TJ Flinn I Zelenetz AD Burger JA. A Phase 2 Study of Idelalisib Plus Rituximab in Treatment-Naive Older Patients With Chronic Lymphocytic Leukemia. Blood (2015) 126(25):2686–94. doi: 10.1182/blood-2015-03-630947 PMC4732760 26472751

[168] Stilgenbauer S Eichhorst B Schetelig J Coutre S Seymour JF Munir T. Venetoclax in Relapsed or Refractory Chronic Lymphocytic Leukaemia With 17p Deletion: A Multicentre, Open-Label, Phase 2 Study. Lancet Oncol (2016) 17(6):768–78. doi: 10.1016/S1470-2045(16)30019-5 27178240

[169] Byrd JC Furman RR Coutre SE Burger JA Blum KA Coleman M. Three-Year Follow-Up of Treatment-Naive and Previously Treated Patients With CLL and SLL Receiving Single-Agent Ibrutinib. Blood (2015) 125:2497–506. doi: 10.1182/blood-2014-10-606038 PMC4400288 25700432

[170] Farooqui MZ Valdez J Martyr S Aue G Saba N Niemann CU. Ibrutinib for Previously Untreated and Relapsed or Refractory Chronic Lymphocytic Leukaemia with TP53 Aberrations: A Phase 2, Single-Arm Trial. Lancet Oncol (2015) 16: (2):169–76. doi: 10.1016/S1470-2045(14)71182-9 PMC4342187 25555420

[171] Coutre SE Barrientos JC Brown JR de Vos S Furman RR Keating MJ. Management of Adverse Events Associated With Idelalisib Treatment: Expert Panel Opinion. Leuk Lymphoma (2015) 56(10):2779–86. doi: 10.3109/10428194.2015.1022770 PMC4732460 25726955

[172] Roberts AW Davids MS Pagel JM Kahl BS Puvvada SD Gerecitano JF. Targeting BCL2 With Venetoclax in Relapsed Chronic Lymphocytic Leukemia. N Engl J Med (2016) 374(4):311–22. doi: 10.1056/NEJMoa1513257 PMC7107002 26639348

[173] Seymour JF Ma S Brander DM Choi MY Barrientos J Davids MS. Venetoclax Plus Rituximab in Relapsed or Refractory Chronic Lymphocytic Leukaemia: A Phase 1b Study. Lancet Oncol (2017) 18(2):230–40. doi: 10.1016/S1470-2045(17)30012-8 PMC5316338 28089635

[174] Visentin A Gurrieri C Imbergamo S Lessi F di Maggio SA Frezzato F. Epidemiology and Risk Factors of Invasive Fungal Infections in a Large Cohort of Patients With Chronic Lymphocytic Leukemia. Hematol Oncol (2017) 35:925–8. doi: 10.1002/hon.2343 27641225

[175] Kotton CN. Poznanski MC Vaccination of Oncology Patients: An Effective Tool and an Opportunity Not to be Missed. Oncologist (2012) 17(1):1–2. doi: 10.1634/theoncologist.2011-0383 PMC3267807 22240542

[176] Righi E Gallo T Azzini AM Mazzaferri F Cordioli M Merighi M. Review of Vaccinations in Adult Patients With Secondary Immunodeficiency. Infect Dis Ther (2021) 10(2):637–61. doi: 10.1007/s40121-021-00404-y PMC7941364 33687662

[177] Calaminus G Hence B Laws HJ Groeger M MacKenzie CR Göbel U. Diphtheria (D) and Tetanus (T) Antibody Values in Children With Acute Lymphoblastic Leukaemia (ALL) After Treatment According to Co-ALL 05/92. Klin Padiatr (2007) 219(6):355–60. doi: 10.1055/s-2007-990290 18050047

[178] Mauro FR Giannarelli D Galluzzo CM Vitale C Visentin A Riemma C. Response to the Conjugate Pneumococcal Vaccine (PCV13) in Patients With Chronic Lymphocytic Leukemia (CLL). Leukemia (2021) 35:737–46. doi: 10.1038/s41375-020-0884-z 32555297

[179] Tsigrelis C Ljungman P. Vaccinations in Patients With Hematological Malignancies. Blood Rev (2016) 30(2):139–47. doi: 10.1016/j.blre.2015.10.001 26602587

[180] Rubin LG Levin MJ Ljungman P Davies EG Avery R Tomblyn M. IDSA Clinical Practice Guideline for Vaccination of the Immunocompromised Host. Clin Infect Dis (2013) 58(3):309–18. doi: 10.1093/cid/cit816 24421306

[181] Pasiarski M Rolinski J Grywalska E Stelmach-Goldys A Korona- Glowniak I Gozdz S. Antibody and Plasmablast Response to 13-Valent Pneumococcal Conjugate Vaccine in Chronic Lymphocytic Leukemia Patients – Preliminary Report. PloS One (2014) 9(12):e114966. doi: 10.1371/journal.pone.0114966 25506837 PMC4266633

[182] Sinisalo M Aittoniemi J Oivanen P Kayhty H Olander RM Vilpo J. Response to Vaccination Against Different Types of Antigens in Patients With Chronic Lymphocytic Leukaemia. Br J Haematol (2001) 114(1):107–10. doi: 10.1046/j.1365-2141.2001.02882.x 11472353

[183] Sinisalo M Aittoniemi J Kayhty H Vilpo J. Vaccination Against Infections in Chronic Lymphocytic Leukemia. Leuk Lymphoma (2003) 44(4):649–52. doi: 10.1080/1042819031000063408 12769342

[184] Pollyea DA Brown JM Horning SJ. Utility of Influenza Vaccination for Oncology Patients. J Clin Oncol (2010) 28(14):2481–90. doi: 10.1200/JCO.2009.26.6908 20385981

[185] Ferry BL Misbah SA Stephens P Sherrell Z Lythgoe H Bateman E. Development of an Anti-*Salmonella Typhi* Vi ELISA: Assessment of Immunocompetence in Healthy Donors. Clin Exp Immunol (2004) 136(2):297–303. doi: 10.1111/j.1365-2249.2004.02439.x 15086394 PMC1809015

[186] Sinisalo M Aittoniemi J Kayhty H Vilpo J. *Haemophilus Influenzae* Type B (Hib) Antibody Concentrations and Vaccination Responses in Patients With Chronic Lymphocytic Leukaemia: Predicting Factors for Response. Leuk Lymphoma (2002) 43(10):1967–9. doi: 10.1080/1042819021000015916 12481893

[187] Brenol CV da Mota LM Cruz BA Pileggi GS Pereira IA Rezende LS. Brazilian Society of Rheumatology Consensus on Vaccination of Patients With Rheumatoid Arthritis. Rev Bras Reumatol (2012) 53(1):4–23. doi: 10.1016/S2255-5021(13)70002-6 23588512

[188] Navarrete MA Heining-Mikesch K Schuler F Bertinetti-Lapatki C Ihorst G Keppler-Hafkemeyer A. Upfront Immunization With Autologous Recombinant Idiotype Fab Fragment Without Prior Cytoreduction in Indolent B-Cell Lymphoma. Blood (2011) 117(5):1483–91. doi: 10.1182/blood-2010-06-292342 21045197

[189] Vallejo AN Weyand CM. Goronzy JJ T-Cell Senescence: A Culprit of Immune Abnormalities in Chronic Inflammation and Persistent Infection. Trends Mol Med (2004) 10(3):119–24. doi: 10.1016/j.molmed.2004.01.002 15102354

[190] Goronzy JJ Fulbright JW Crowson CS Poland GA O’Fallon WM Weyand CM. Value of Immunological Markers in Predicting Responsiveness to Influenza Vaccination in Elderly Individuals. J Virol (2001) 75(24):12182–7. doi: 10.1128/JVI.75.24.12182-12187.2001 PMC116115 11711609

[191] Biggar RJ Chaturvedi AK Goedert JJ Engels EA. AIDS-Related Cancer and Severity of Immunosuppression in Persons With AIDS. J Natl Cancer Inst (2007) 99(12):962–72. doi: 10.1093/jnci/djm010 17565153

[192] Grulich AE van Leeuwen MT Falster MO. Vajdic CM Incidence of Cancers in People With HIV/AIDS Compared With Immunosuppressed Transplant Recipients: A Meta-Analysis. Lancet (2007) 370(9581):59–67. doi: 10.1016/S0140-6736(07)61050-2 17617273

[193] Alemu A Richards JO Oaks MK. Thompson MA Vaccination in Multiple Myeloma: Review of Current Literature. Clin Lymphoma Myeloma Leuk (2016) 16:495–502. doi: 10.1016/j.clml.2016.06.006 27364264

[194] Bladé J Rosiñol L. Renal, Hematologic and Infectious Complications in Multiple Myeloma. Best Pract Res Clin Haematol (2005) 18:635–52. doi: 10.1016/j.beha.2005.01.013 16026742

[195] Bae J Smith R Daley J Mimura N Tai YT Anderson KC. Myeloma-Specific Multiple Peptides Able to Generate Cytotoxic T Lymphocytes: A Potential Therapeutic Application in Multiple Myeloma and Other Plasma Cell Disorders. Clin Cancer Res (2012) 18:4850–60. doi: 10.1158/1078-0432.CCR-11-2776 PMC3839582 22753586

[196] Singhal S Metha J. Reimmunization After Blood or Marrow Stem Cell Transplantation. Bone Marrow Transplant (1999) 23:637–46. doi: 10.1038/sj.bmt.1701640 10218839

[197] CDC (Centers for disease control and prevention). Guidelines for Preventing Opportunistic Infections Among Hematopoietic Stem Cell Transplant Recipients. MMMR (2000) 49:1–128. 11718124

[198] Nordøy T Kolstad A Endresen PC Holte H Kvaløy S Kvalheim G. Persistent Changes in the Immune System 4–10 Years After ABMT. Bone Marrow Transplant (1999) 24:873–8. doi: 10.1038/sj.bmt.1702006 10516699

[199] Nordøy T Husebekk A Aaberge IS Jenum PA Samdal HH Flugsrud LB. Humoral Immunity to Viral and Bacterial Antigens in Lymphoma Patients 4-10 Years After High-Dose Therapy With ABMT. Serological Responses to Revaccinations According to EBMT Guidelines. Bone Marrow Transplant (2001) 28(7):681–7. doi: 10.1038/sj.bmt.1703228.2017 11704791

[200] Bharucha T Ming D Breuer JA. Critical Appraisal of ‘Shingrix’, a Novel Herpes Zoster Subunit Vaccine (HZ/Su or GSK1437173A) for Varicella Zoster Virus. Hum Vaccin Immunother (2017) 13(8):1789–97. doi: 10.1080/21645515.2017.1317410 PMC5557227 28426274

[201] Rapoport AP Stadtmauer EA Aqui N Badros A Cotte J Chrisley L. Restoration of Immunity in Lymphopenic Individuals With Cancer by Vaccination and Adoptive T-Cell Transfer. Nat Med (2005) 11(11):1230–7. doi: 10.1038/nm1310 16227990

[202] Bhakta N Liu Q Ness KK Baassiri M Eissa H Yeo F. The Cumulative Burden of Surviving Childhood Cancer: An Initial Report From the St Jude Lifetime Cohort Study (SJLIFE). Lancet (2017) 390(10112):2569–82. doi: 10.1016/S0140-6736(17)31610-0 PMC5798235 28890157

[203] Maas HA Janssen-Heijnen ML Olde Rikkert MG Machteld Wymenga AN. Comprehensive Geriatric Assessment and its Clinical Impact in Oncology. Eur J Cancer (2007) 43:2161–9. doi: 10.1016/j.ejca.2007.08.002 17855074

[204] Wiernik PH. Second Neoplasm in Patients With Chronic Lymphocytic Leukemia. Curr Treat Options Oncol (2004) 5:215–23. doi: 10.1007/s11864-004-0013-7 15115650

[205] Hisada M Biggar RJ Greene MH Fraumeni JF Jr Travis LB. Solid Tumors After Chronic Lymphocytic Leukemia. Blood (2001) 98:1979 – 1981. doi: 10.1182/blood.V98.6.1979 11535538

[206] Molica S. Second Neoplasms in Chronic Lymphocytic Leukemia: Incidence and Pathogenesis With Emphasis on the Role of Different Therapies. Leuk Lymphoma (2005) 46(1):49–54. doi: 10.1080/10428190400007524 15621780

[207] Engels EA Frisch M Goedert JJ Biggar RJ Miller RW. Merkel Cell Carcinoma and HIV Infection. Lancet (2002) 359:497–8. doi: 10.1016/S0140-6736(02)07668-7 11853800

[208] Popp S Waltering S Herbst C Moll I Boukamp P. UV-B-Type Mutations and Chromosomal Imbalances Indicate Common Pathways for the Development of Merkel and Skin Squamous Cell Carcinomas. Int J Cancer (2002) 99:352–60. doi: 10.1002/ijc.10321 11992403

[209] Hodgson NC. Merkel Cell Carcinoma: Changing Incidence Trends. J Surg Oncol (2005) 89:1–4. doi: 10.1002/jso.20167 15611998

[210] Feng H Shuda M Chang Y Moore PS. Clonal Integration of a Polyomavirus in Human Merkel Cell Carcinoma. Science (2008) 319:1096–100. doi: 10.1126/science.1152586 PMC2740911 18202256

[211] Brewer JD Shanafelt TD Otley CC Roenigk RK Cerhan JR Kay NE. Chronic Lymphocytic Leukemia is Associated With Decreased Survival of Patients With Malignant Melanoma and Merkel Cell Carcinoma in a SEER Population-Based Study. J Clin Oncol (2012) 30:843–9. doi: 10.1200/JCO.2011.34.9605 22331952

[212] Tseng YD Nguyen MH Baker K Cook M Redman M Lachance K. Effect of Patient Immune Status on the Efficacy of Radiation Therapy and Recurrence-Free Survival Among 805 Patients With Merkel Cell Carcinoma. Int J Radiat Oncol Biol Phys (2018) 102(2):330–9. doi: 10.1016/j.ijrobp.2018.05.075 PMC9592535 30191867

[213] Russo AL Chen YH Martin NE Vinjamoori A Luthy SK Freedman A. Low-Dose Involved-Field Radiation in the Treatment of non-Hodgkin Lymphoma: Predictors of Response and Treatment Failure. Int J Radiat Oncol Biol Phys (2013) 86:121–7. doi: 10.1016/j.ijrobp.2012.12.024 23414765

[214] Arroyo AB Fernández-Pérez MP Del Monte A Águila S Méndez R Hernández-Antolín R. miR-146a is a Pivotal Regulator of Neutrophil Extracellular Trap Formation Promoting Thrombosis. Haematologica (2021) 106(6):1636–46. doi: 10.3324/haematol.2019.240226 PMC8168495 32586906

[215] Campos J Ponomaryov T De Prendergast A Whitworth K Smith CW Khan AO. Neutrophil Extracellular Traps and Inflammasomes Cooperatively Promote Venous Thrombosis in Mice. Blood Adv (2021) 5(9):2319–24. doi: 10.1182/bloodadvances.2020003377 PMC8114549 33938940

[216] Chechlinska M Kowalewska M Nowak R. Systemic Inflammation as a Confounding Factor in Cancer Biomarker Discovery and Validation. Nat Rev Cancer (2010) 10:2–3. doi: 10.1038/nrc2782 20050335

[217] Kim TY Gu J-Y Jung HS Koh Y Kim I. Kim HK Elevated Extracellular Trap Formation and Contact System Activation in Acute Leukemia. J Thromb Thrombolysis (2018) 46:379–85. doi: 10.1007/s11239-018-1713-3 30099724

[218] Podaza E Sabbione F Risnik D Borge M Almejún MB Colado A. Neutrophils From Chronic Lymphocytic Leukemia Patients Exhibit an Increased Capacity to Release Extracellular Traps (NETs). Cancer Immunol Immunother (2016) 66:77–89. doi: 10.1007/s00262-016-1921-7 27796477 PMC11029506

[219] Erpenbeck L. Schön MP Neutrophil Extracellular Traps: Protagonists of Cancer Progression? Oncogene (2017) 36:2483–90. doi: 10.1038/onc.2016.406 27941879

[220] Demers M Krause DS Schatzberg D Martinod K Voorhees JR Fuchs TA. Cancers Predispose Neutrophils to Release Extracellular Dna Traps That Contribute to Cancer-Associated Thrombosis. Proc Natl Acad Sci USA (2012) 109:13076–81. doi: 10.1073/pnas.1200419109 PMC3420209 22826226

[221] Ramsey PC Rubin RH Tolkoff-Rubin NE Cosimi AB Russell PS Greene R. The Renal Transplant Patient With Fever and Pulmonary Infiltrates: Etiology, Clinical Manifestations, and Management. Med (Baltimore) (1980) 59:206–22. doi: 10.1097/00005792-198005000-00003 6997674

[222] Fan CQ Crawford JM. Sinusoidal Obstruction Syndrome (Hepatic Veno-Occlusive Disease). J Clin Exp Hepatol (2014) 4(4):332–46. doi: 10.1016/j.jceh.2014.10.002 PMC4298625 25755580

[223] Contreras I Arruabarrena C Figueroa MS. Treatment of Bilateral Candidal Endophthalmitis With Intravenous Caspofungin. Retin cases Brief Rep (2007) 1(3):175–7. doi: 10.1097/01.ICB.0000279654.16358.00 25390788

[224] Madney Y Shalaby L Elanany M Adel N Nasr E Alsheshtawi K. Clinical Features and Outcome of Hepatosplenic Fungal Infections in Children With Haematological Malignancies. Mycoses (2020) 63(1):30–7. doi: 10.1111/myc.13002 31514231

[225] Das T Dave VP Dogra A Joseph J Sharma S EMS working group. Endophthalmitis Management Study. Report 1. Protocol Indian J Ophthalmol (2021) 69(7):1936–41. doi: 10.4103/ijo.IJO_199_21 PMC8374761 34146061

[226] Dave VP Pathengay A Nishant K Pappuru RR Sharma S Narayanan R. Clinical Presentations, Risk Factors, and Outcome of Ceftazidime- Resistant Gram-Negative Endophthalmitis. Clin Exp Ophthalmol (2017) 45:254–60. doi: 10.1111/ceo.12833 27616274

[227] Neudorfer M Barrnea Y Geyer O Siegman-Igra Y. Retinal Lesions in Septicemia. Am J Ophthalmol (1993) 116:728–34. doi: 10.1016/S0002-9394(14)73473-7 8250076

[228] Krishna R Amuh D Lowder CY Gordon SM Adal KA Hall G. Should All Patients With Candidaemia Have an Ophthalmic Examination to Rule Out Ocular Candidiasis? Eye (2000) 14:30–4. doi: 10.1038/eye.2000.7 10755096

[229] Henderson DK Edwards JEJ Montgomerie JZ. Hematogenous Candida Endophthalmitis in Patients Receiving Parenteral Hyperalimentation Fluids. J Infect Dis (1981) 143:655–61. doi: 10.1093/infdis/143.5.655 6787141

[230] Mehrad B Paciocco G Martinez FJ Ojo TC Iannettoni MD Lynch JP III. Spectrum of Aspergillus Infection in Lung Transplant Recipients: Case Series and Review of the Literature. Chest (2001) 119:169–75. doi: 10.1378/chest.119.1.169 11157600

[231] Al-Alawi A Ryan CF Flint JD Muller NL. Aspergillus-Related Lung Disease. Can Respir J (2005) 12:377–87. doi: 10.1155/2005/759070 16307029

[232] Karnak D Avery RK Gildea TR Sahoo D Mehta AC. Endobronchial Fungal Disease: An Under-Recognized Entity. Respiration (2007) 74:88–104. doi: 10.1159/000094708 16864987

[233] Stoller JK Picker LJ Weiss ST Thurer RL Kasdon EJ. Pulmonary Artery-Bronchial Fistula Complicating Chronic Lymphocytic Leukemia. Chest (1994) 86(1):134–5. doi: 10.1378/chest.86.1.134 6734273

[234] Bortin MM Kay HEM Gale RP Rimm AA. Factors Associated With Interstitial Pneumonitis After Bone-Marrow Transplantation for Acute Leukaemia. Lancet (1982) 1:437–9. doi: 10.1016/S0140-6736(82)91633-6 6121103

[235] Rosenow EC 3rd Wilson WR Cockerill FR 3rd. Pulmonary Disease in the Immunocompromised Host. 1. Mayo Clin Proc (1985) 60(7):473–87. doi: 10.1016/s0025-6196(12)60872-6 2409412

[236] Mitchell R Nivison-Smith I Anazodo A Tiedemann K Shaw PJ Teague L. Outcomes of Haematopoietic Stem Cell Transplantation for Inherited Metabolic Disorders: A Report From the Australian and New Zealand Children’s Haematology Oncology Group and the Australasian Bone Marrow Transplant Recipient Registry. Pediatr Transplant (2013) 17(6):582–8. doi: 10.1111/petr.12109 23802616

[237] Adeyemo TA Adeyemo WL Adediran A Akinbami AA. Akanmu AS Orofacial Manifestation of Hematological Disorders: Hemato-Oncologic and Immuno-Deficiency Disorders. Indian J Dent Res (2011) 22:688–97. doi: 10.4103/0970-9290.93458 22406715

[238] Bhambal AM Shrivastava H Naik SP Nair P Saawarn N. Oral Manifestations of Systemic Leukemia-First Sign of Presentation. J Indian Soc Periodontol (2021) 25(4):347–9. doi: 10.4103/jisp.jisp_551_20 PMC8336771 34393407

[239] Sydney SB Serio F. Acute Monocytic Leukemia Diagnosed in a Patient Referred Because of Gingival Pain. J Am Dent Assoc (1981) 103:886–9. doi: 10.14219/jada.archive.1981.0403 6947009

[240] Brenneise CV Mattson JS Commers JR. Acute Myelomonocytic Leukemia With Oral Manifestations: Report of a Case. J Am Dent Assoc (1988) 117:835–7. doi: 10.14219/jada.archive.1988.0125 3204244

[241] Cammarata-Scalisi F Girardi K Strocchio L Merli P Garret-Bernardin A Galeotti A. Oral Manifestations and Complications in Childhood Acute Myeloid Leukemia. Cancers (Basel) (2020) 12(6):1634. doi: 10.3390/cancers12061634 PMC7352340 32575613

[242] Rinaggio J Neiders ME Aguirre A Kumar V. Using Immunofluorescence in the Diagnosis of Chronic Ulcerative Lesions of the Oral Mucosa. Compend Contin Educ Dent (1999) 20(10):943–4. 10650375

[243] Declerck F Vinckier F. Oral Complications of Leukemia. Quintessence Int (1988) 19:717–28. 3077686

[244] Barrett AP. Oral Changes as Initial Diagnostic Indicators in Acute Leukemia. J Oral Med (1986) 41:234–8. 3465925

[245] Duffy JH Driscoll EJ. Oral Manifestations of Leukemia. Oral Surg Oral Med Oral Pathol (1985) 11:484–90. doi: 10.1016/0030-4220(58)90092-6 13553299

[246] Takagi M Sakota Y Ishikawa G Kamiyama R Nakajima T Nomura T. Oral Manifestations of Acute Promyelocytic Leukemia. J Oral Maxillofac Surg (1978) 36:589–93. 277651

[247] Jones AC Bentsen TY Freedman PD. Mucormycosis of the Oral Cavity. Oral Surg Oral Med Oral Pathol (1993) 75:455–60. doi: 10.1016/0030-4220(93)90170-9 8464609

[248] Barrett AP. A Long-Term Prospective Clinical Study of Oral Complications During Conventional Chemotherapy for Acute Leukemia. Oral Surg Oral Med Oral Pathol (1987) 63:313–6. doi: 10.1016/0030-4220(87)90196-4 3495768

[249] Blomgren J Back H. Oral Hairy Leukoplakia in a Patient With Multiple Myeloma. Oral Surg Oral Med Oral Pathol Oral Radiol Endod (1996) 82:408–10. doi: 10.1016/S1079-2104(96)80305-2 8899778

[250] Perusse R. Oral Candidiasis and Multiple Myeloma: An Unusual Association. Oral Surg Oral Med Oral Pathol (1994) 78:264–6. doi: 10.1016/0030-4220(94)90157-0 7936599

[251] Reinish EI Raviv M Srolovitz H Gornitsky M. Tongue, Primary Amyloidosis, and Multiple Myeloma. Oral Surg Oral Med Oral Pathol (1994) 77:121–5. doi: 10.1016/0030-4220(94)90272-0 8139827

[252] Yasunaga JI Matsuoka M. Oncogenic Spiral by Infectious Pathogens: Cooperation of Multiple Factors in Cancer Development. Cancer Sci (2018) 109(1):24–32. doi: 10.1111/cas.13443 29143406 PMC5765297

[253] Gangemi S Allegra A Musolino C. Lymphoproliferative Disease and Cancer Among Patients With Common Variable Immunodeficiency. Leuk Res (2015) 39(4):389–96. doi: 10.1016/j.leukres.2015.02.002 25711943

[254] Bohnhorst J Rasmussen T Moen SH Fløttum M Knudsen L Børset M. Toll-Like Receptors Mediate Proliferation and Survival of Multiple Myeloma Cells. Leukemia (2006) 20:1138–44. doi: 10.1038/sj.leu.2404225 16617319

[255] Jego G Bataille R Geffroy-Luseau A Descamps G Pellat-Deceunynck C. Pathogen-Associated Molecular Patterns are Growth and Survival Factors for Human Myeloma Cells Through Toll-Like Receptors. Leukemia (2006) 20:1130–7. doi: 10.1038/sj.leu.2404226 16628189

[256] Chiron D Pellat-Deceunynck C Amiot M Bataille R Jego G. TLR3 Ligand Induces NF-{Kappa}B Activation and Various Fates of Multiple Myeloma Cells Depending on IFN-{Alpha} Production. J Immunol (2009) 182(7):4471–8. doi: 10.4049/jimmunol.0803113 19299748

[257] Kovacs E. How Does Interleukin-6 Affect the Membrane Expressions of Interleukin-6 Receptor and Gp130 and the Proliferation of the Human Myeloma Cell Line OPM-2? BioMed Pharmacother (2003) 57:489–94. doi: 10.1016/j.biopha.2003.08.024 14637393

[258] Dankbar B Padró T Leo R Feldmann B Kropff M Mesters RM. Vascular Endothelial Growth Factor and Interleukin-6 in Paracrine Tumor-Stromal Cell Interactions in Multiple Myeloma. Blood (2000) 95:2630. doi: 10.1182/blood.V95.8.2630 10753844

[259] Hersey P Wotherspoon J Reid G Gunz FW. Hypogammaglobulinaemia Associated With Abnormalities of Both B and T Lymphocytes in Patients With Chronic Lymphatic Leukaemia. Clin Exp Immunol (1980) 39(3):698–707. 6445798 PMC1538113

[260] Sánchez-Ramón S Dhalla F Chapel H. Challenges in the Role of Gammaglobulin Replacement Therapy and Vaccination Strategies for Hematological Malignancy. Front Immunol (2016) 7:317. doi: 10.3389/fimmu.2016.00317 27597852 PMC4993076

[261] Allegra A Imbesi C Bitto A Ettari R. Drug Repositioning for the Treatment of Hematologic Disease: Limits, Challenges and Future Perspectives. Curr Med Chem (2021) 28(11):2195–217. doi: 10.2174/0929867327999200817102154 33138750

[262] Tian F Wang C Tang M Li J Cheng X Zhang S. The Antibiotic Chloramphenicol may be an Effective New Agent for Inhibiting the Growth of Multiple Myeloma. Oncotarget (2016) 7(32):51934–42. doi: 10.18632/oncotarget.10623 PMC5239525 27437770

[263] Moriya S Che X-F Komatsu S Abe A Kawaguchi T Gotoh A. Macrolide Antibiotics Block Autophagy Flux and Sensitize to Bortezomib *via* Endoplasmic Reticulum Stress-Mediated CHOP Induction in Myeloma Cells. Int J Oncol (2013) 42:1541–50. doi: 10.3892/ijo.2013.1870 PMC3661227 23546223

[264] Qiu XH Shao JJ Mei JG Li HQ Cao HQ. Clarithromycin Synergistically Enhances Thalidomide Cytotoxicity in Myeloma Cells. Acta Haematol (2016) 135:103. doi: 10.1159/000438855 26505646

[265] Herishanu Y Avivi I Aharon A Shefer G Levi S Bronstein Y. Efficacy of the BNT162b2 mRNA COVID-19 Vaccine in Patients With Chronic Lymphocytic Leukemia. Blood (2021) 137(23):3165–73. doi: 10.1182/blood.2021011568 PMC8061088 33861303

[266] Allegra A Penna G Innao V Greve B Maisano V Russo S. Vaccination of Multiple Myeloma: Current Strategies and Future Prospects. Crit Rev Oncol Hematol (2015) 96(2):339–54. doi: 10.1016/j.critrevonc.2015.06.003 26123319

[267] Fuchs TA Abed U Goosmann C Hurwitz R Schulze I Wahn V. Novel Cell Death Program Leads to Neutrophil Extracellular Traps. J Cell Biol (2007) 176:231–41. doi: 10.1083/jcb.200606027 PMC2063942 17210947

